# Holistic Impact of CKD: A Clinical, Economic, and Environmental Analysis by IMPACT CKD

**DOI:** 10.1016/j.ekir.2025.03.051

**Published:** 2025-04-02

**Authors:** Naveen Rao, Francisco Brotons-Munto, Ana F. Moura, Janwillem W.H. Kocks, Ming-hui Zhao, Steven Chadban, Hannah Guiang, Stacey Priest, Stephen Brown

**Affiliations:** 1 BioPharmaceuticals Medical, AstraZeneca, Cambridge, UK; 2 Trinitat Health Center, Valencia, Spain; 3 Escola Bahiana de Medicina e Saúde Pública, Salvador, Brazil; 4 General Practitioners Research Institute, Groningen, The Netherlands; 5 Observational and Pragmatic Research Institute, Singapore, Singapore; 6 Groningen Research Institute Asthma and COPD, University of Groningen, University Medical Center Groningen, Groningen, The Netherlands; 7 Department of Pulmonology, University of Groningen, University Medical Center Groningen, Groningen, The Netherlands; 8 Peking University First Hospital, Beijing, China; 9 Royal Prince Alfred Hospital, Camperdown, New South Wales, Australia; 10 EVERSANA, Burlington, Ontario, Canada

**Keywords:** chronic kidney disease, kidney replacement therapy, microsimulation epidemiology model, multidimensional burden, policy

## Abstract

**Introduction:**

Chronic kidney disease (CKD) is a growing global health problem driven by aging and the rise of comorbidities, including diabetes and hypertension. Although clinical and economic burdens have been reported, there is a lack of information on the multidimensional impact of CKD across countries with varying demographics and health system archetypes.

**Methods:**

The validated IMPACT CKD model was used to project the clinical, health care resource use (HCRU), economic, patient, societal, and environmental burden of CKD in 8 countries (Australia, Brazil, China, Germany, Netherlands, Spain, United Kingdom, and United States) over 10 years (baseline: 2022; simulated years: 2023–2032).

**Results:**

By 2032, 11.7% to 16.5% of the countries’ populations were projected to have CKD, with prevalence rising in 7 countries over 10 years, and a shift toward later stages. These increases were accompanied by projected increases of 3.6% to 170.8% and 41.9% to 80.1% in the number of patients living on dialysis and with a functioning kidney transplant, respectively. Driven by this growing clinical burden, increases of over 19% in CKD costs and 20% in kidney replacement therapy (KRT) costs were projected across all countries. Impaired work productivity led to significant projected losses in gross domestic product, full-time equivalents, and tax revenue. Freshwater consumption, fossil fuel depletion, and carbon production in patients on KRT were projected to increase by over 11% in all the countries.

**Conclusion:**

This study demonstrates the multifaceted burden of CKD globally and supports the adoption of policies such as CKD screening programs and public health awareness to promote early diagnosis and treatment to mitigate the growing disease burden.

## Graphical abstract

**Figure:**
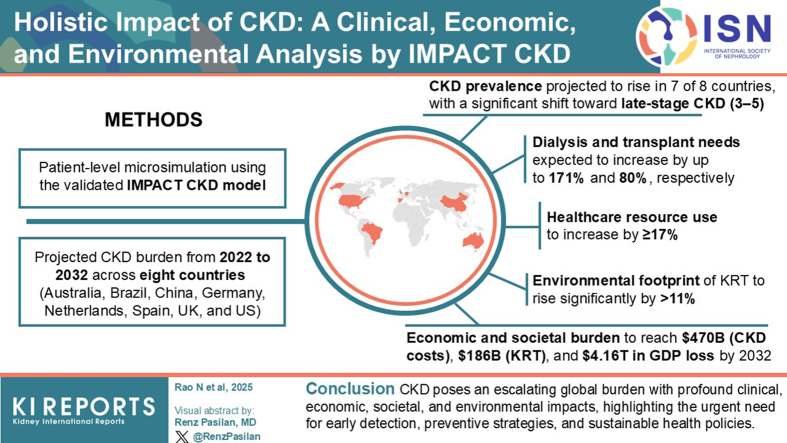

CKD is a prevalent health issue, affecting 13% of the global population, with an escalating economic and environmental impact.^1^ Its multifaceted burden, propelled by comorbidities and aging populations,^2^ is expected to position CKD as the fifth leading cause of death in 2040.^3^^,^^4^ Despite its prevalence, CKD often remains undiagnosed, leading to delayed interventions and missed opportunities to prevent CKD progression and complications.^5^^,^^6^

The significant CKD burden on patients, society, health care systems, and the environment increases with advancing stages.^3^^,^^7^, ^8^, ^9^ Health-related quality of life is reduced across the spectrum of CKD with the greatest reductions seen in more advanced stages, particularly end-stage kidney disease.^8^^,^^10^, ^11^, ^12^ Cardiovascular (CV) disease is a leading cause of morbidity and mortality among patients with CKD, particularly among patients receiving KRT by dialysis^13^^,^^14^ or kidney transplantation.^15^ The management of CKD and its related complications contributes to significant health care costs and resource use.^6^^,^^16^ Health care expenditures for patients with end-stage kidney disease who require KRT with dialysis or transplantation consume an estimated 2% to 3% of the annual health care budget in high-income countries.^17^ The environmental impact of KRT is considerable because of carbon production associated with its high water consumption, use of single-use plastic, and high energy expenditures.^18^

Presently, there are major discrepancies between the increasing CKD burden and the global recognition of CKD as a health priority. Only 51% of governments worldwide recognize CKD as a health priority; only 34% of countries have CKD-specific policy plans.^19^ To effectively reduce CKD burden, policymakers need to evaluate the impact of national strategies on a long-term timescale, using a comprehensive viewpoint that captures the dynamic patient, societal, and environmental burdens.^20^ The IMPACT CKD model was developed to analyze CKD burden from clinical, HCRU, economic, patient, societal, and environmental perspectives.^21^ IMPACT CKD is the first model to consider these burdens using a disease framework developed by the London School of Economics and validated by over 60 global cross-functional experts.

Although previous studies have explored CKD’s epidemiologic and economic facets,^1^^,^^3^^,^^7^ a gap remains in understanding its multidimensional burden across countries with varying demographics, health system financing, and access-to-care. Our study, utilizing the IMPACT CKD microsimulation model, aims to fill this gap by quantifying and projecting the global impact of CKD from 2022 to 2032, offering insights on the global trends and country-specific impacts of CKD for health care policymakers, practitioners, and patients.

## Methods

### Model Overview

The IMPACT CKD model is a patient-level microsimulation designed to simulate CKD development and progression to estimate the clinical, HCRU, economic, patient, societal, and environmental burden within a population.^21^ The model is composed of the following 3 modules: (i) population generation using prevalence distributions; (ii) patient journey simulation for 1 million individuals (baseline year [2022] and 10 simulated years [2023–2032]); and (iii) data aggregation and reporting extrapolated to the population of interest.^21^ The disease model generates a cohort representative of the population of interest (including individuals with and without CKD) and considers baseline estimated glomerular filtration rate (eGFR) and albuminuria level, eGFR rate of decline, acute kidney injury, CV events (stroke and myocardial infarction), comorbidities (heart failure, hypertension, and diabetes), and dialysis and kidney transplant. Disease states include non-CKD, CKD stages 1, 2, 3a, 3b, 4, 5 (predialysis and conservative care), dialysis, kidney transplant, and death. Stages are defined by eGFR and urine albumin-to-creatinine ratio as per the Kidney Disease: Improving Global Outcomes guidelines.^22^ Individuals were assigned to an access-to-care class (e.g., none, partial, or unlimited) to reflect differences in health care systems.

In each 1-year cycle, CKD progression, transplant, initiation of dialysis, and clinical events could occur. Current demographic and health care characteristics inform the output of clinical burden which, in turn, drives resource use, economic, patient, societal, and environmental burden of CKD. Additional details on model methodology and key model assumptions can be found in the Supplementary Methods.

**Figure 1:**
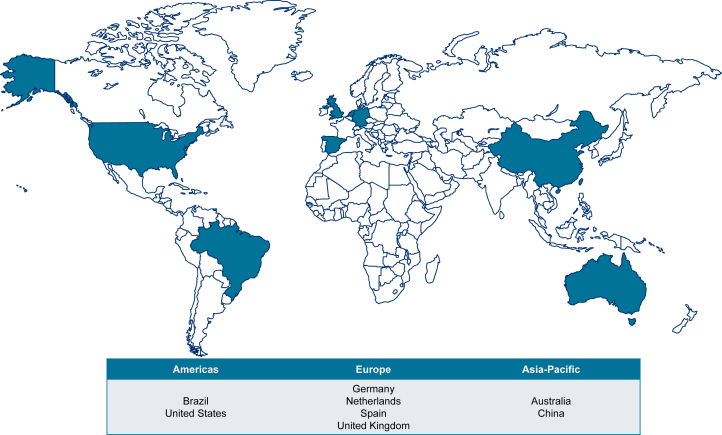
Countries included in the IMPACT CKD microsimulation.

In the present analyses, 8 country populations (Australia, Brazil, China, Germany, The Netherlands, Spain, United Kingdom, and United States) were simulated (Figure 1). These countries represent a broad geographic area with various health care system archetypes to provide insights into the global CKD burden. Data availability was also considered.

Further, during model development, several validation steps were conducted in line with guidelines.^23^, ^24^, ^25^, ^26^ Face validity of the model structure was tested with clinical experts from the 8 countries, as well as technical validation (i.e., independent review and extreme value testing). Calibration and external predictive validation were conducted for every country to confirm that the projected values for model outcomes are aligned with known data. Validation targets were prioritized for key prevalence and/or incidence rates, including CKD stage distribution and KRT at baseline, as well as yearly incidence of clinical events and mortality in each country (Supplementary Table S1A and B).

#### Model Inputs

Country-specific data were leveraged from recent projects, including INSIDE CKD^20^ (epidemiology inputs and costs), DISCOVER CKD^27^ (eGFR decline equations and HCRU), and PACE CKD^28^ (financial well-being and burden inputs).^29^ To supplement the data derived from these sources, country-specific literature searches and environmental research^29^ were conducted. Published data, within the last 10 years, was prioritized for use in the analyses; however, older data were used if there was limited availability. If no country- or region-specific sources were available, suitable, expert-validated country proxies were used. Data input sources are summarized in Supplementary Table S2.

#### Sensitivity Analyses

In addition to the primary disease burden analyses for each country, sensitivity analyses were conducted for key model drivers identified during model development. These included the following 4 sensitivity analyses for parameters that directly impact the number of patients with CKD. For all sensitivity analyses conducted for each country, the parameters were changed independently as described whereas all other inputs remained at their default base case values.

1. Increase birth or immigration rate by 10% and decrease mortality inputs by 10%.
2. Decrease birth or immigration rate by 10% and increase mortality inputs by 10%.
3. Increase annual eGFR decline in the general (non-CKD) population by 10%.
4. Decrease annual eGFR decline in the general (non-CKD) population by 10%.

## Results

The results are presented by the following burden domains: (i) clinical impact, (ii) HCRU, (iii) economic impact, (iv) patient impact, (v) societal impact, and (vi) environmental impact. Global trends and country-specific characteristics are presented below.

### Clinical Impact

#### Baseline Projections and Prevalence

At baseline (2022), population characteristics and clinical prevalence differed across countries (Supplementary Table S3). Germany had the largest proportion of patients aged ≥ 65 years (22.2%), China had the largest proportion of patients with CKD in stage 1 to 2 (69.0%), and the United States had the highest prevalence of dialysis and kidney transplant (1871.0 per million population and 833.0 per million population, respectively).

#### Projected Changes in CKD Prevalence and Number of Patients

**Figure 2:**
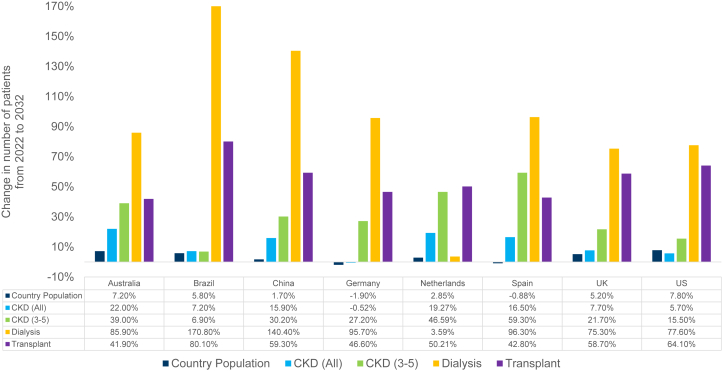
Change in country population, number of patients with CKD, receiving dialysis, and living post-transplant from 2022 to 2032. CKD, chronic kidney disease; UK, United Kingdom; US, United States.

By 2032, CKD prevalence would range between 11.7% and 16.5% of the population in the 8 countries and increase in 7 of the 8 countries (Table 1). In the United States, CKD prevalence was projected to decline from a baseline of 16.9% (2022) to 16.5% (2032). Despite CKD cases increasing similarly to other countries, prevalence would slightly decrease because of the greater growth of the United States’ population. Further, the number of patients with CKD was predicted to increase by 2032 for all countries except Germany, where a decrease of 0.5% was projected. The percentage change in the number of patients with CKD, patients receiving dialysis, and patients living with a functioning kidney transplant between 2022 and 2032 are presented in Figure 2. The number of patients with CKD, late-stage CKD (3–5), receiving dialysis, living posttransplant, and experiencing CV events (myocardial infarction and stroke) are included in Table 1.

**Table 1:** Clinical outcomes in patients with CKD from 2022 to 2032

Outcome	Year	Australia	Brazil	China	Germany	The Netherlands	Spain	UK	US
Overall prevalence									
CKD (all)	2022	10.3%	12.1%	12.3%	13.9%	12.5%	13.1%	12.4%	16.9%
	2032	11.7%	12.2%	14.0%	14.1%	14.5%	15.4%	12.7%	16.5%
Number of patients and percentage change in the number of patients from 2022 to 2032									
CKD (all)	2022	2.67 M	25.83 M	175.0 M	11.70 M	2.20 M	6.20 M	8.27 M	55.00 M
	2032	3.25 M	27.68 M	202.7 M	11.64 M	2.63 M	7.23 M	8.91 M	58.11 M
	2022 to 2032	22.0%	7.2%	15.9%	-0.5%	19.3%	16.5%	7.7%	5.7%
CKD (3–5)	2022	866.29 K	11.86 M	54.29 M	4.52 M	916.51 K	2.19 M	3.54 M	22.09 M
	2032	1.20 M	12.68 M	70.67 M	5.75 M	1.34 M	3.49 M	4.30 M	25.51 M
	2022 to 2032	39.0%	6.9%	30.2%	27.2%	46.6%	59.3%	21.7%	15.5%
Receiving dialysis	2022	14.9 K	136.5 K	770.0 K	94.1 K	6.9 K	30.8 K	33.1 K	610.3 K
	2032	27.8 K	369.7 K	1.9 M	184.1 K	7.1 K	60.5 K	58.0 K	1.1 M
	2022 to 2032	85.9%	170.8%	140.4%	95.7%	3.6%	96.3%	75.3%	77.6%
Living posttransplant	2022	13.3 K	63.7 K	192.5 K	48.6 K	12.4 K	38.0 K	40.3 K	271.7 K
	2032	18.8 K	114.7 K	306.6 K	71.3 K	18.6 K	54.3 K	63.9 K	445.9 K
	2022 to 2032	41.9%	80.1%	59.3%	46.6%	50.2%	42.8%	58.7%	64.1%
Number of CV events and the percentage change in the number of events from 2022 to 2032									
MI events	2022	10.5 K	68.2 K	342.2 K	82.4 K	17.7 K	18.9 K	36.2 K	200.6 K
	2032	11.6 K	73.1 K	399.3 K	87.1 K	25.1 K	23.8 K	38.9 K	222.5 K
	2022 to 2032	9.6%	7.2%	16.7%	5.7%	41.7%	25.9%	7.4%	10.9%
Stroke events	2022	13.4 K	119.2 K	914.0 K	36.0 K	2.4 K	27.6 K	28.8 K	185.0 K
	2032	23.3 K	169.1 K	1.4 M	45.4 K	3.9 K	41.1 K	36.3 K	280.5 K
	2022 to 2032	73.5%	41.9%	57.1%	26.2%	63.5%	49.2%	26.0%	51.7%

CKD, chronic kidney disease; CV, cardiovascular; K, thousand; M, million; MI, myocardial infarction; UK, United Kingdom; US, United States.

#### Projected Changes in Late Stage (3–5) CKD

The number of patients in later CKD stages (3–5) was projected to increase from 2022 to 2032 in all 8 countries, reaching nearly 125 million people by 2032. Increases exceeding 30% in Australia, China, The Netherlands, and Spain were projected. The highest increase was predicted in Spain (59.3%). In contrast, the smallest increase was predicted for Brazil (6.9%).

#### Projected Changes in Dialysis and Transplant

Over the next decade, the number of patients requiring dialysis was expected to increase by 3.6% to 170.8% in all 8 countries (Figure 2 and Table 1). The highest increase in the number of patients requiring dialysis was projected in Brazil (170.8%), and the smallest increase was projected in The Netherlands (3.6%). The number of patients living with a functioning transplant was projected to grow by >40% (range: 41.9%–80.1%) in all 8 countries. The greatest increase in the number of patients with kidney transplant was projected for Brazil (80.1%), and the smallest increase was projected for Australia (41.9%). An estimated additional 2.4 million patients were projected to require KRT by 2032.

#### CKD Prevalence Sensitivity Analysis Projections

Across the countries, the sensitivity analyses generally resulted in trends consistent with the base case analyses with differences in magnitude as expected. Minor increases were observed in CKD prevalence in 2032 with the birth or immigration (+10%) and mortality inputs (−10%) analysis (sensitivity 1) and eGFR decline in the general population (+10%) analysis (sensitivity 3), and minor decreases with the birth or immigration (−10%) and mortality inputs (+10%) analysis (sensitivity 2) and eGFR decline in the general population (−10%) analysis (sensitivity 4) as expected compared with the base case primary analyses (Supplementary Figure S1). This was due to analyses 1 and 3 resulting in increases in the number of patients with CKD through reduced death (sensitivity 1) and increased progression from non-CKD to CKD (sensitivity 3). The opposite effect occurred for analyses 2 and 4. Similarly, there were slightly greater percentage increases in the number of all patients with CKD and stage 3 to 5 from 2022 to 2032 with analyses 1 and 3, and smaller increases with analyses 2 and 4 compared with the base case for all countries (Supplementary Figures S2 and S3). Furthermore, there were generally greater percentage increases in the number of patients living on dialysis and posttransplant with analysis 1 and smaller percentage increases with analysis 2 compared with the base case analyses. No changes in dialysis or transplant projections were observed with analyses 3 and 4 because of these analyses impacting the number of patients in earlier rather than later CKD stages directly (Supplementary Figures S4 and S5).

Overall, the clinical burden associated with CKD was projected to increase or remain high in all 8 countries. The CKD prevalence was projected to increase in 7 of the 8 countries, with an alarming increase in late stage (3–5) CKD in all 8 countries. As a result, demand for KRT resources would increase subsequently over the next decade.

#### CV Event Projections

The number of CV events among people with CKD was predicted to increase in all countries over the next decade. The highest increase in myocardial infarction events was projected for The Netherlands (41.7% increase from 17,696 events in 2022 to 25,084 events in 2032). The projected increases in myocardial infarction events were lowest for Germany (5.7% increase from 82,361 events in 2022 to 87,082 in 2032). Stroke events were expected to increase by >25% in all 8 countries (range: 26.0%–73.5%), with the highest increase projected for Australia (73.5% increase from 13,405 cases in 2022 to 23,251 cases in 2032). The percentage change and projected number of patients by CV events are presented in Table 1.

### HCRU

**Figure 3:**
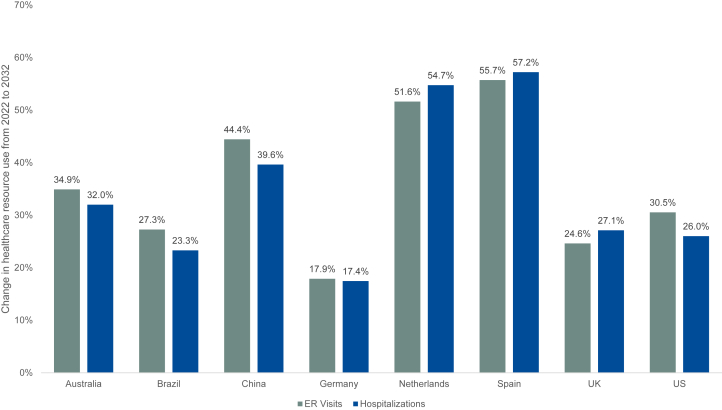
Change in health care resource use from 2022 to 2032. ER, emergency room; UK, United Kingdom; US, United States.

Because CKD prevalence and its progression to late-stage CKD were projected to increase globally, a corresponding increase in HCRU is projected within the 8 countries between 2022 and 2032. Emergency room (ER) visits and hospitalizations were predicted to increase by over 20% among patients diagnosed with CKD within 7 of the 8 countries (Table 2 and Figure 3). Germany, the only country with an increase < 20%, had projected increases in ER visits and hospitalizations of 17.9% and 17.4%, respectively. The highest increases in HCRU were projected for The Netherlands and Spain. For The Netherlands, ER visits and hospitalizations were predicted to increase by 51.6% and 54.7%, respectively. For Spain, ER visits and hospitalizations were predicted to increase by 55.7% and 57.2%, respectively.

**Table 2:** Number of diagnosed patients with CKD with ER visits and hospitalizations in 2022 and 2032

Outcome	Year	Australia	Brazil	China	Germany	The Netherlands	Spain	UK	US
ER visits	2022	262.9 K	1.4 M	5.9 M	1.4 M	244.9 K	667.8 K	974.4 K	2.7 M
	2032	354.6 K	1.8 M	8.5 M	1.6 M	371.3 K	1.0 M	1.2 M	3.5 M
	2022 to 2032	34.9%	27.3%	44.4%	17.9%	51.6%	55.7%	24.6%	30.5%
Hospitalizations	2022	485.1 K	16.5 M	68.5 M	2.6 M	422.5 K	1.2 M	1.7 M	31.1 M
	2032	640.3 K	20.4 M	95.7 M	3.1 M	653.8 K	1.9 M	2.2 M	39.2 M
	2022 to 2032	32.0%	23.3%	39.6%	17.4%	54.7%	57.2%	27.1%	26.0%

CKD, chronic kidney disease; ER, emergency room; K, thousand; M, million; UK, United Kingdom; US, United States.

### Economic Impact

**Figure 4:**
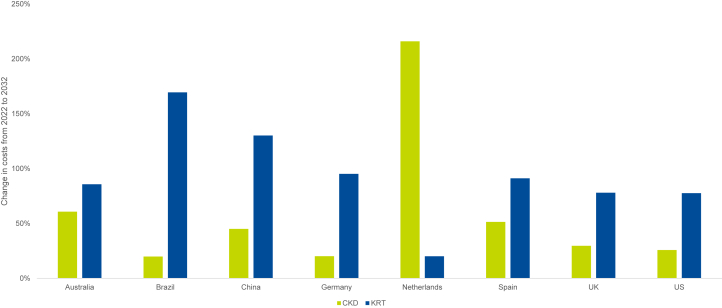
Change in CKD stage 1-5 (non-KRT) and KRT costs from 2022 to 2032. CKD, chronic kidney disease; KRT, kidney replacement therapy; UK, United Kingdom; US, United States.

Given projected increases in prevalence of CKD, and late-stage CKD in particular, a substantial increase in associated health care system costs is also projected (Table 3 and Figure 4). The highest increase in CKD (excluding KRT) costs, associated with patients diagnosed with CKD, was projected for The Netherlands (216.0%), from €682.53 million ($717.69 million United States Dollars [USDs]) in 2022 to €2.16 billion ($2.27 billion USD) in 2032. The lowest increase was projected for Brazil (19.9%), from R$24.89 billion ($4.82 billion USD) in 2022 to R$29.84 billion ($5.78 billion USD) in 2032. Across the 8 countries, the economic toll of CKD (i.e., diagnosed patients with CKD stage 3–5 [non-KRT]) is estimated to reach $470 billion USD by 2032.

**Table 3:** CKD and KRT costs in 2022 and 2032

Outcome	Year	Australia	Brazil	China	Germany	The Netherlands	Spain	UK	US
CKD costs	2022	$2.65 B	R$24.89 B	¥1.14 T	€12.23 B	€682.53 M	€3.41 B	£1.89 B	$149.89 B
	2032	$4.26 B	R$29.84 B	¥1.66 T	€14.69 B	€2.16 B	€5.17 B	£2.45 B	$188.57 B
	2022 to 2032	60.8%	19.9%	45.0%	20.1%	216.0%	51.4%	29.6%	25.8%
KRT costs	2022	$1.63 B	R$6.35 B	¥88.46 B	€5.04 B	€628.38 M	€1.88 B	£1.09 B	$75.12 B
	2032	$3.02 B	R$17.12 B	¥203.61 B	€9.84 B	€754.85 M	€3.59 B	£1.95 B	$133.43 B
	2022 to 2032	85.8%	169.5%	130.2%	95.2%	20.1%	91.2%	78.0%	77.6%

B, billion; CKD, chronic kidney disease; KRT, kidney replacement therapy; M, million; T, trillion; UK, United Kingdom; US, United States.

KRT costs were projected to increase by over 20% in all 8 countries (Table 3 and Figure 4). Increased KRT costs were driven by the number of patients with late-stage CKD (Table 1). The highest increase in KRT costs were projected for Brazil. In Brazil, KRT costs were predicted to increase by 169.5%, from R$6.35 billion ($1.23 billion USD) in 2022 to R$17.12 billion ($3.32 billion USD) in 2032. Across the 8 countries, the economic toll of KRT is estimated to reach $186 billion USD by 2032.

### Patient Impact

Projected growth in the number of patients with late-stage CKD was predicted to result in an increase in symptom burden, reduced quality of life and an increase in CV events. The model predicted the average quality-adjusted life-years, a measure of disease burden, including both the quality and quantity of life lived, of patients with CKD over the next decade (range: 7.096 [Germany]–7.889 [Australia]) (Table 4).

**Table 4:** QALYs per patient with CKD and financial burden over the 10-Year model time horizon

Outcome	Year	Australia	Brazil	China	Germany	The Netherlands	Spain	UK	US
QALYs per patient with CKD	Total Over 10-Years	7.889	7.685	7.884	7.096	7.516	7.752	7.385	7.417
Financial Burden NDD/DD^a^	Total Over 10-Years	26.54/ 21.08	22.92/ 23.44	22.92/ 23.44	23.41/ 26.91	26.54/21.08	26.54/ 21.08	26.54/ 21.08	22.92/ 23.44

CKD, chronic kidney disease; DD, dialysis dependent; NDD, nondialysis dependent; QALY, quality-adjusted life-year; UK, United Kingdom; US, United States.

a FACIT-COST score ranges from 0 to 44. Lower scores signify higher financial toxicity, or a higher financial burden.

Furthermore, the financial burden, assessed by the 44-point FACIT-cost score,^30^ projected for patients with CKD was high across the 8 countries. In patients receiving dialysis, financial burden ranged from 21.08 (Australia, The Netherlands, Spain, United Kingdom) to 26.91 (Germany). A higher score generally implies better financial well-being.^30^ For context, an employed person with at least 1 chronic illness in the United States has a mean FACIT-Cost score of 28.5, compared with their unemployed counterpart with a mean FACIT-Cost score of 21.0.^31^ The cumulative lost income for patients with CKD and caregivers by patient absenteeism (i.e., missing work due to symptoms or appointments) or presenteeism and caregiver absenteeism (i.e., missing work to care for a patient with CKD) ranged from €6.4 billion ($6.8 billion USD), in The Netherlands, to $413.4 billion USD in the United States. Costs associated with lost income because of caregiver absenteeism represent a substantial proportion, ranging from €215.6 million ($227 million USD), in The Netherlands, to $14.9 billion USD in the United States. Overall, the cumulative lost income from presenteeism and absenteeism of patients and caregivers across the 8 countries was estimated to reach $835 billion USD.

### Societal Impact

Beyond the direct health care costs, there was an alarming socioeconomic impact because of CKD. The projected increase in missed workdays for patients with CKD and for caregivers was substantial over 10 years. Across the 8 countries, by 2032, there were 2.85 billion cumulative missed workdays because of patient absenteeism and 319.15 million missed workdays because of caregiver absenteeism projected. In addition, 8.56 billion missed workdays because of presenteeism were estimated to occur in patients with CKD across the 8 countries.

These productivity impairments were predicted to result in considerable societal impacts, including lost gross domestic product, full-time equivalents, and tax revenue over the 10-year model time horizon (Table 5). Across the 8 countries, 10-year productivity losses were equated to 48.8 million full-time equivalent workers (Supplementary Figure S6), resulting in a loss of $4.16 trillion USD in gross domestic product (Supplementary Figure S7), and $37 billion USD in lost tax revenue, which could affect government budgets and health care services (Supplementary Figure S8). The need for health policy strategies to address this high socioeconomic burden of CKD is underscored.

**Table 5:** Missed workdays, lost GDP, lost FTEs, and lost tax revenue over the 10-Year model time horizon

Outcome	Australia	Brazil	China	Germany	Netherlands	Spain	UK	US
Missed workdays because of absenteeism in patients with CKD	22.1 M	360.4 M	1.5 B	71.9 M	16.5 M	34.4 M	89.1 M	743.4 M
Missed workdays because of presenteeism in patients with CKD	66.0 M	1.1 B	4.6 B	204.9 M	48.0 M	97.1 M	263.2 M	2.2 B
Missed workdays because of absenteeism in caregivers of patients with CKD	3.3 M	40.4 M	142.1 M	5.4 M	2.2 M	6.7 M	10.0 M	109.1 M
Lost GDP in diagnosed patients with CKD and caregivers (local currency and USD)	$71.3 B	R$1.3 T	¥5.0 T	€226.6 B	€50.0 B	€104.7 B	£279.8 B	$2.4 T
	$49.4 B^a^	$242.4 B^a^	$741.0 B^a^	$238.2 B^a^	$52.6 B^a^	$110.1 B^a^	$345.0 B^a^	
Lost FTEs in diagnosed patients with CKD and Caregivers	400.9 K	4.6 M	26.8 M	1.6 M	351.1 K	630.9 K	1.8 M	12.7 M
Lost tax revenue because of diagnosed patients with CKD and caregiver absenteeism (local currency and USD)	$458.3 M	R$8.7 B	¥65.1 B	€3.4 B	€321.4 M	€1.1 B	£2.1 B	$17.4 B
	$0.3 B^a^	$1.7 B^a^	$9.7 B^a^	$3.6 B^a^	$0.3 B^a^	$1.1 B^a^	$2.6 B^a^	

B, billion; CKD, chronic kidney disease; FTE, full-time equivalent; GDP, gross domestic product; K, thousand; KRT, kidney replacement therapy; M, million; OECD, Organisation for Economic Co-operation and Development; T, trillion; UK, United Kingdom; US, United States; USD, US dollar.

a Currency conversions performed using the national currency per USD in 2022 from OECD, available from: https://stats.oecd.org/index.aspx?queryid=169. Values used in conversions were as follows: Australia, 1.442; Brazil, 5.164, China, 6.737; Germany, The Netherlands, Spain, 0.951; UK, 0.811.

### Environmental Impact

**Figure 5:**
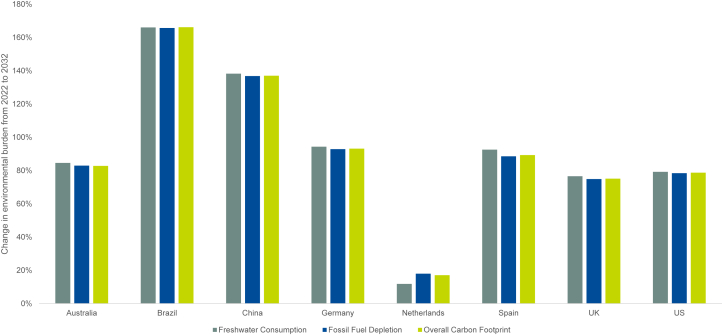
Change in environmental burden in KRT patients from 2022 to 2032. KRT, kidney replacement therapy; UK, United Kingdom; US, United States.

In addition to its well-documented clinical and economic challenges, CKD presents substantial environmental concerns, particularly in the context of KRT because of the high resource use among patients receiving dialysis. Significant increases in all 3 environmental metrics were projected from patients receiving KRT across all countries because of the large increase in the number of patients receiving KRT (Figure 5).

In Supplementary Table S4, we present the detailed environmental burden projections associated with KRT. Across the 8 countries, freshwater consumption in 2032 was projected to be 440.3 million m^3^, equivalent to the annual water usage of approximately 2.7 million households.^32^ Fossil fuel use in 2032 was projected to be 11.2 billion kg oil eq., which is equivalent to the annual power for 148.8 million 100-watt lightbulbs running 24 hours a day.^33^ Carbon footprint in 2032 was projected to be 29.1 billion kg carbon dioxide, equivalent to the annual carbon dioxide emissions of approximately 17.3 million cars.^34^ The results highlight the often-overlooked ecological dimensions of CKD treatment, underscoring the need for environmentally sustainable approaches in health care.

## Discussion

IMPACT CKD is the first model to consider clinical, HCRU, economic, patient, societal, and environmental burdens, using a disease framework that encompasses the CKD pathway across the continuum of care. In the present IMPACT CKD analysis, CKD burden was quantified in 2022 and projected to 2032 across 8 countries. The results of the microsimulation provide key insights into the multidimensional burden of CKD. In efforts to mitigate the global CKD burden to patients, caregivers, and health systems, these results provide actionable targets to increase awareness and inform health care policy.

Across all 8 countries, the projected increases in advanced CKD and KRT are accompanied by substantial increases in HCRU, health care costs, patient, societal, and environmental burdens. Growth in HCRU associated with patients with diagnosed CKD from 2022 to 2032 ranged from 17% to 58% across the 8 countries. High levels of financial burden were projected over 10 years for all patients with CKD, where patients requiring dialysis experienced slightly higher financial burden as nondialysis patients indicating that CKD management, across all stages, increases the patient’s economic burden. In addition, the projected impact of CKD on productivity was substantial. The projected number of missed workdays because of absenteeism and presenteeism in patients with CKD and absenteeism in caregivers is a critical aspect of CKD’s projected burden, presenting a societal challenge for patients and caregivers alike. This loss of productivity is reflected by substantial reductions in the projected gross domestic product, full-time equivalents, and tax revenue. Such losses are potentially preventable with policies to mitigate the rising CKD burden. The environmental analyses reveal a large increase in freshwater consumption, fossil fuel depletion, and carbon dioxide emissions in patients receiving KRT across all countries from 2022 to 2032, emphasizing the need to reduce disease progression to KRT and for investment in sustainable technologies for health care practices.

Variation in CKD trends were expected between the countries because of differences in population characteristics, dynamics, and health care systems. The model uses hundreds of inputs that interact to influence epidemiologic changes, disease development, disease progression, and KRT patterns. Therefore, model results cannot easily be traced to a few causative inputs. However, age distribution, eGFR and albuminuria distribution (determinants of CKD stage), eGFR decline, population growth, mortality rates, comorbidity prevalence, and rates of dialysis and kidney transplant are critical inputs that greatly influence the model results and can vary widely by country. For context, as demonstrated by the sensitivity analyses, mortality and rate of CKD development directly impact the magnitude of the projections, albeit trends remain consistent. Furthermore, many of the European countries (e.g., Germany, The Netherlands, and Spain) have age distributions skewed toward older groups, whereas Brazil and China have age distributions with higher proportions of people aged < 65 years. Countries with older populations are likely to have higher proportions of their population with low eGFR and albuminuria that will impact the projected growth of CKD prevalence. Population prevalence of comorbidities associated with CKD has a major impact on CKD projections, as is the case in the United States. Because diabetes, hypertension, and heart failure have detrimental impacts on kidney function, increases in prevalence of these comorbid conditions will likely increase CKD prevalence.

Germany is the only country where the number of patients with CKD was predicted to decline slightly over the next decade (−0.5%). This decrease is likely driven by the large proportion of the 2022 German population aged ≥ 65 years coupled with a declining population size. It is plausible that Germany may have already reached peak CKD prevalence within their population. As expected, the sensitivity analyses reveal minor deviations in the number of patients with CKD compared with the changes over the time horizon with the primary analysis because of the direct effects on the entire population size and number of patients with CKD. Despite the overall CKD population declining slightly with the primary analysis, the model still predicted a 27.2% increase in CKD stage 3 to 5 patients in Germany, reflecting disease progression. Furthermore, growth in patient numbers receiving KRT was not always aligned with growth in CKD prevalence, because of usage patterns for dialysis and transplant. The United States and some European countries share high rates of historical incident dialysis and transplant, whereas Brazil has lower incidence rates but higher rates of growth in the past decade (Supplementary Table S5). These usage patterns may provide some explanation for the large increase in dialysis usage in Brazil despite a smaller increase in the number of patients diagnosed with CKD. The large increase in the number of patients undergoing dialysis in Brazil also aligns with recent publications^35^ and may reflect an inefficiency in the early diagnosis and management of the disease. Moreover, the relatively stagnant number of prevalent dialysis patients over the time horizon in The Netherlands differs from other countries where larger projected increases are observed. This is most likely driven by a high rate of timely living donor transplantation, as well as the utilization of kidney exchange programs in The Netherlands, reducing the need for dialysis treatment.^36^

Differences observed across countries in projections for HCRU, economic, patient, societal, and environmental burden domains are in part attributable to differences in the clinical burden as discussed above (e.g., number and stage of patients with CKD). Notably, in The Netherlands, CKD costs were predicted to increase to a greater extent than in the other 7 countries, driven by a larger increase in diagnosed stage 4 patients (881.5%) (Supplementary Table S6). It is conceivable that the number of patients diagnosed with stage 4 CKD increase to such a large level because of the low baseline of patients with stage 4 CKD (2022; 0.62%/10,748), the slightly lower mortality rate in patients with stage 4 CKD in the first year (1.81%), and a relatively large number of patients with stage 3 CKD at baseline who may transition to stage 4 over the model time horizon. At baseline, China had a low percentage of patients with CKD in stage 4 at baseline and projects to have large increases in stage 4 CKD over the time horizon (+505.5%). In addition, China had a low proportion of patients receiving KRT among all patients with CKD, contributing to a lower proportion (11%) of the overall CKD-related costs in 2032. In contrast, in other countries, KRT costs account for a large proportion of the overall CKD-related costs, with KRT accounting for 28% of the overall economic toll by 2032 across all countries.

The limitations of the IMPACT CKD model are common to all microsimulation analyses. The analyses require large amounts of data inputs collected across a range of studies from different timepoints which differ in population, quality, and methodology. Because comprehensive, longitudinal datasets capturing all required inputs are unrealistic, data uncertainty is unavoidable because of differences between data sources within and across countries. Notably, although 2022 is used as the baseline year, model input data have been sourced from other years because of the lack of data availability in the published literature. Proxies were used from similar countries in cases where country-specific data were not available, potentially increasing outcome uncertainty. The model structure reflects the best currently available knowledge of CKD; therefore, gaps in the current knowledge may have introduced uncertainty. Furthermore, CKD prevalence estimates included both diagnosed and undiagnosed patients; however, studies that estimate the proportion of undiagnosed CKD by stage are limited in number and quality, and as such contribute to uncertainty in the field of CKD research. In addition, the projected HCRU (ER visits and hospitalizations) is likely further underestimated because of the focus solely on the inpatient setting. In Europe, United States, and Australia, CKD is often managed in outpatient settings. Lastly, the projected HCRU, economic, patient, societal, and environmental burdens are reflective of patients with diagnosed CKD and are therefore likely underestimated for CKD overall.

Overall, the model results support a CKD epidemic that will grow substantially over the next decade because of changes in age distribution and prevalence of comorbid diabetes and hypertension within each country. The projected increase in advanced CKD and KRT signifies a forthcoming surge in HCRU, with significant financial, societal, and environmental implications for patients, caregivers, and health care systems. These trends suggest a looming public health challenge, necessitating urgent policy interventions in efforts to improve patient conditions and societal outcomes. This study’s findings advocate for policies aimed at early diagnosis and effective interventions to slow disease progression, in turn reducing the burdens and costs associated with late-stage CKD. Future studies will project the changes in overall CKD burden in response to screening and treatment strategies. The IMPACT CKD model will also be used to project CKD burden across additional countries in the future.

In conclusion, the IMPACT CKD model serves as an evidence-based decision-making tool with the potential to help shape future policy and practice in the detection and management of CKD. Through its comprehensive and multidimensional analysis, the model has revealed key findings about the future trajectory of CKD. In this IMPACT CKD analysis, the growing clinical, HCRU, economic, patient, societal, and environmental burden was quantified for 2022 and projected to 2032, providing insight into the global holistic burden of CKD. The predicted increases in advanced CKD and KRT are expected to have immense impact on patients, caregivers, society, health care systems, and the environment. These projections underscore and highlight the growing challenges posed by CKD and the urgent need for transformative policy measures to mitigate its impact. The IMPACT CKD model outcomes strongly advocate for proactive policy that emphasizes early detection and appropriate management of CKD to mitigate the extensive burdens of late-stage CKD revealed by this study. These changes are imperative for improving patient outcomes and critical in reducing the overarching impact on health care systems and society. Insights from this model and its capabilities to analyze the effect of potential policy measures, such as targeted screening or treatment compliance, should guide future policies to effectively reduce the global burden of CKD.

## Disclosure

NR is employed at AstraZeneca and holds stocks in AstraZeneca. FB-M has received funding from AstraZeneca for their involvement in the present study, is part of semFYC, from which he has received payment for lectures or presentations, and participates on a Data Safety Monitoring Board or Advisory Board at AstraZeneca and Boehringer Ingelheim. AFM has received funding from AstraZeneca for their involvement in the present study. JWHK's institution has received grants from AstraZeneca, Boehringer Ingelheim, Chiesi Pharmaceuticals, GSK, TEVA, MSD, COVIS Pharma, Mundi Pharma, Valneva, G3P, and Eli Lilly; consulting payments from AstraZeneca, Boehringer Ingelheim, Chiesi Pharmaceuticals, GSK, TEVA, MSD, COVIS Pharma, and Jansen; and lecture or presentations payments from Mundi Pharma, ALK-Albello, Chiesi Pharmaceuticals, Boehringer Ingelheim, Merck, Esculaap Media, TEVA, AstraZeneca, and Sanofi. JWHK is a director of the General Practitioners Research Institute, president and board director of the International Primary Care Respiratory Group, member of CAHAG Scientific Committee, board member of Inhalation Institute Netherlands, a GINA advocate for GINA, and hold stocks in the General Practitioners Research Institute and Lothar Medtec. M-hZ has received honoraria and advisory fees from AstraZeneca, GSK, Roche, SanReno, Novartis, BeiGene, Everest Medicines, Zailab, and Eli Lilly. SC has received speaker payments from AstraZeneca, GSK, Boehringer Ingelheim, and Novartis; payment for advisory board participation from CSL-Vifor, Novartis, and Alexion; and advises the Australian Government on kidney transplantation and chronic kidney disease. HG, SP, and SB are employed at EVERSANA, which received funding from AstraZeneca to conduct the study, and has received support for attending meetings and/or travel by AstraZeneca.

## Supplementary Material

Supplementary File (PDF) **Supplementary Methods. Supplementary References. Figure S1.** Sensitivity analysis projections - prevalence of CKD (all) in 2032. **Figure S2.** Sensitivity analysis projections - percentage change in number of CKD (all) patients from 2022 to 2032. **Figure S3.** Sensitivity analysis projections - percentage change in number of CKD (3-5) patients from 2022 to 2032. **Figure S4.** Sensitivity analysis projections - percentage change in number of patients receiving dialysis from 2022 to 2032. **Figure S5.** Sensitivity analysis projections - percentage change in number of patients living post-transplant from 2022 to 2032. **Figure S6.** Ten-year lost FTEs in diagnosed patients with CKD and caregivers. **Figure S7.** Ten-year lost GDP in diagnosed patients with CKD and caregivers. **Figure S8.** Ten-year lost tax revenue due to diagnosed patients with CKD and caregiver absenteeism. **Table S1.** (A) Model values and validation targets by country (Australia, Brazil, China, Germany). (B) Model values and validation targets by country (Netherlands, Spain, UK, US). **Table S2.** Summary of data inputs by country. **Table S3.** Summary of population characteristics at baseline (2022) by country. **Table S4.** Predicted freshwater consumption, fossil fuel depletion, and overall carbon footprint from KRT patients in 2022 and 2032. **Table S5.** Growth rate of incident dialysis from 2022 to 2032**. Table S6.** Percentage of patients with CKD and the number of diagnosed patients in CKD stages 3 and 4, annual mortality rate in stage 4, and yearly stage 4 cost input.

## Footnotes

Supplementary File (PDF)

**Supplementary Methods.**

**Supplementary References.**
**Figure S1.** Sensitivity analysis projections - prevalence of CKD (all) in 2032. **Figure S2.** Sensitivity analysis projections - percentage change in number of CKD (all) patients from 2022 to 2032. **Figure S3.** Sensitivity analysis projections - percentage change in number of CKD (3-5) patients from 2022 to 2032. **Figure S4.** Sensitivity analysis projections - percentage change in number of patients receiving dialysis from 2022 to 2032. **Figure S5.** Sensitivity analysis projections - percentage change in number of patients living post-transplant from 2022 to 2032. **Figure S6.** Ten-year lost FTEs in diagnosed patients with CKD and caregivers. **Figure S7.** Ten-year lost GDP in diagnosed patients with CKD and caregivers. **Figure S8.** Ten-year lost tax revenue due to diagnosed patients with CKD and caregiver absenteeism. **Table S1.** (A) Model values and validation targets by country (Australia, Brazil, China, Germany). (B) Model values and validation targets by country (Netherlands, Spain, UK, US) **Table S2.** Summary of data inputs by country. **Table S3.** Summary of population characteristics at baseline (2022) by country. **Table S4.** Predicted freshwater consumption, fossil fuel depletion, and overall carbon footprint from KRT patients in 2022 and 2032. **Table S5.** Growth rate of incident dialysis from 2022 to 2032. **Table S6.** Percentage of patients with CKD and the number of diagnosed patients in CKD stages 3 and 4, annual mortality rate in stage 4, and yearly stage 4 cost input.
