# Supplementary material for: Holistic Impact of CKD: A Clinical, Economic, and Environmental Analysis by IMPACT CKD
Source: Kidney Int Rep. 2025 Apr 2;10(7):2116–27. doi: 10.1016/j.ekir.2025.03.051 (PMC12266219; doi:10.1016/j.ekir.2025.03.051)
Supplement: Supplementary File (PDF) — Supplementary Methods. Supplementary References. Figure S1. Sensitivity analysis projections - prevalence of CKD (all) in 2032. Figure S2. Sensitivity analysis projections - percentage change in number of CKD (all) patients from 2022 to 2032. Figure S3. Sensitivity analysis projections - percentage change in number of CKD (3-5) patients from 2022 to 2032. Figure S4. Sensitivity analysis projections - percentage change in number of patients receiving dialysis from 2022 to 2032. Figure S5. Sensitivity analysis projections - percentage change in number of patients living post-transplant from 2022 to 2032. Figure S6. Ten-year lost FTEs in diagnosed patients with CKD and caregivers. Figure S7. Ten-year lost GDP in diagnosed patients with CKD and caregivers. Figure S8. Ten-year lost tax revenue due to diagnosed patients with CKD and caregiver absenteeism. Table S1. (A) Model values and validation targets by country (Australia, Brazil, China, Germany). (B) Model values and validation targets by country (Netherlands, Spain, UK, US). Table S2. Summary of data inputs by country. Table S3. Summary of population characteristics at baseline (2022) by country. Table S4. Predicted freshwater consumption, fossil fuel depletion, and overall carbon footprint from KRT patients in 2022 and 2032. Table S5. Growth rate of incident dialysis from 2022 to 2032. Table S6. Percentage of patients with CKD and the number of diagnosed patients in CKD stages 3 and 4, annual mortality rate in stage 4, and yearly stage 4 cost input. [file mmc1.pdf]

Supplementary Materials

Supplementary Methods

Key Methodological Model Assumptions

The following is a list of key methodological model assumptions:

- Due to limited health system capacity and access-to-care, as well as an imbalance between demand and supply of organs, caps on dialysis and transplantation were implemented and aligned with historical incidence data, while accounting for yearly growth rates in dialysis (set to align with historical incident growth data) and transplant (set to align with the growth rate of the country population).
- The model did not differentiate between types of transplants (i.e., living donor, deceased donor, grafts) instead using average data for costs and survival.
- The probability of primary transplant graft failure and subsequent graft failure was not captured in the model (i.e., transplants were assumed to last for the entire time horizon).
- Decline in eGFR is informed by DISCOVER CKD with fast progressors modeled explicitly.[27]
- Interdependence of patient characteristics was not considered due to a lack of comprehensive data that would inform a correlation matrix for all patient characteristics.
- eGFR and albuminuria values were not adjusted for comorbidities due to limitations in the available data.
- Hypothetical individuals entering the model due to births or immigration did not have CKD and would not develop CKD within the 10-year time horizon. This is a simplifying assumption reflecting the low rates in those populations.
- Due to a lack of comprehensive data in patients with CKD, the model did not consider the impact of patient characteristics (except CKD stage), including comorbidities, on the incidence of a clinical event. Instead, rates for patients with CKD were estimated using rates of events were for the general population adjusted by a hazard ratio for each event by CKD stage.
- Patients could only have one of each type of event per cycle. This is a simplifying assumption.
- Clinical events for patients living with a kidney transplant or on dialysis were not simulated.
- Conservatively, the occurrence of a hospitalization due to a HF event was only assigned in patients with HF.
- Costs were not discounted or adjusted for inflation

Supplementary Figures

Supplementary Figure S1: Sensitivity analysis projections - prevalence of CKD (all) in 2032

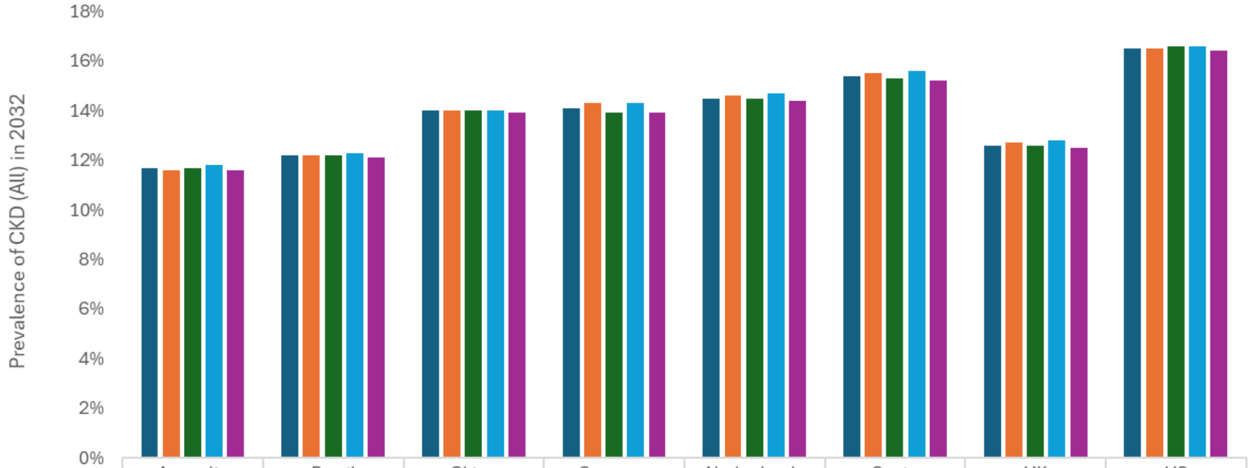

	Australia	Brazil	China	Germany	Netherlands	Spain	UK	US
Base Case	11.7%	12.2%	14.0%	14.1%	14.5%	15.4%	12.6%	16.5%
Sensitivity 1: Birth/Immigration Rate +10% and Mortality Inputs -10%	11.6%	12.2%	14.0%	14.3%	14.6%	15.5%	12.7%	16.5%
Sensitivity 2: Birth/Immigration Rate -10% and Mortality Inputs +10%	11.7%	12.2%	14.0%	13.9%	14.5%	15.3%	12.6%	16.6%
Sensitivity 3: eGFR Decline General Population +10%	11.8%	12.3%	14.0%	14.3%	14.7%	15.6%	12.8%	16.6%
Sensitivity 4: eGFR Decline General Population -10%	11.6%	12.1%	13.9%	13.9%	14.4%	15.2%	12.5%	16.4%

Abbreviations: CKD = chronic kidney disease; eGFR = estimated glomerular filtration rate; UK = United Kingdom; US = United States.

Supplementary Figure S2: Sensitivity analysis projections - percentage change in number of CKD (all) patients from 2022 to 2032

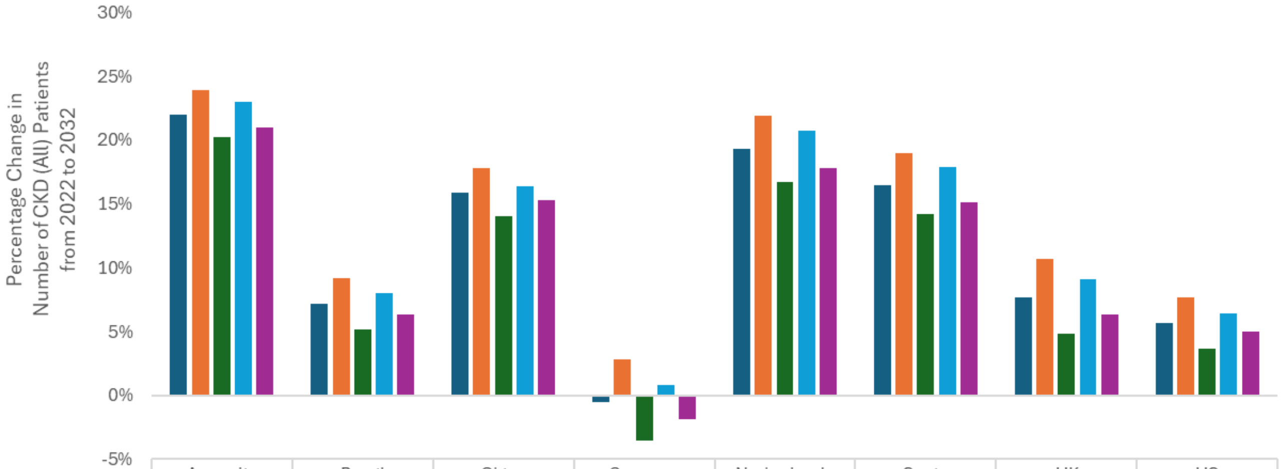

	Australia	Brazil	China	Germany	Netherlands	Spain	UK	US
Base Case	22.0%	7.2%	15.9%	-0.5%	19.3%	16.5%	7.7%	5.7%
Sensitivity 1: Birth/Immigration Rate +10% and Mortality Inputs -10%	23.9%	9.2%	17.8%	2.8%	21.9%	19.0%	10.7%	7.7%
Sensitivity 2: Birth/Immigration Rate -10% and Mortality Inputs +10%	20.2%	5.2%	14.0%	-3.5%	16.7%	14.2%	4.8%	3.7%
Sensitivity 3: eGFR Decline General Population +10%	23.0%	8.0%	16.4%	0.8%	20.7%	17.9%	9.1%	6.4%
Sensitivity 4: eGFR Decline General Population -10%	21.0%	6.3%	15.3%	-1.9%	17.8%	15.1%	6.3%	5.0%

Abbreviations: CKD = chronic kidney disease; eGFR = estimated glomerular filtration rate; UK = United Kingdom; US = United States.

Supplementary Figure S3: Sensitivity analysis projections - percentage change in number of CKD (3-5) patients from 2022 to 2032

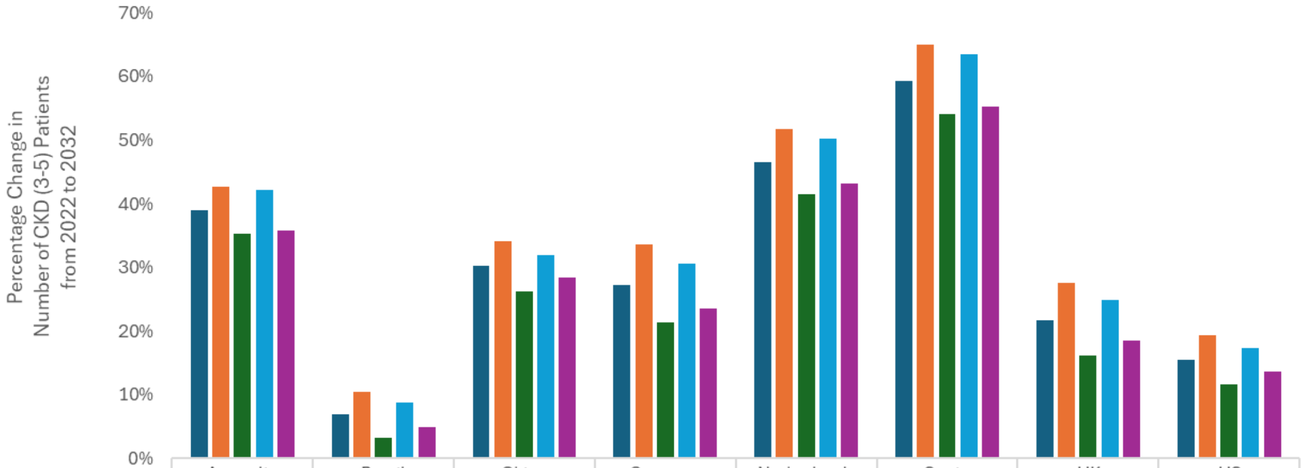

	Australia	Brazil	China	Germany	Netherlands	Spain	UK	US
Base Case	39.0%	6.9%	30.2%	27.2%	46.6%	59.3%	21.7%	15.5%
Sensitivity 1: Birth/Immigration Rate +10% and Mortality Inputs -10%	42.7%	10.5%	34.1%	33.7%	51.8%	65.0%	27.5%	19.4%
Sensitivity 2: Birth/Immigration Rate -10% and Mortality Inputs +10%	35.3%	3.3%	26.3%	21.4%	41.5%	54.0%	16.2%	11.7%
Sensitivity 3: eGFR Decline General Population +10%	42.1%	8.8%	32.0%	30.6%	50.2%	63.4%	24.9%	17.4%
Sensitivity 4: eGFR Decline General Population -10%	35.8%	5.0%	28.4%	23.5%	43.1%	55.2%	18.5%	13.7%

Abbreviations: CKD = chronic kidney disease; eGFR = estimated glomerular filtration rate; UK = United Kingdom; US = United States.

Supplementary Figure S4: Sensitivity analysis projections - percentage change in number of patients receiving dialysis from 2022 to 2032

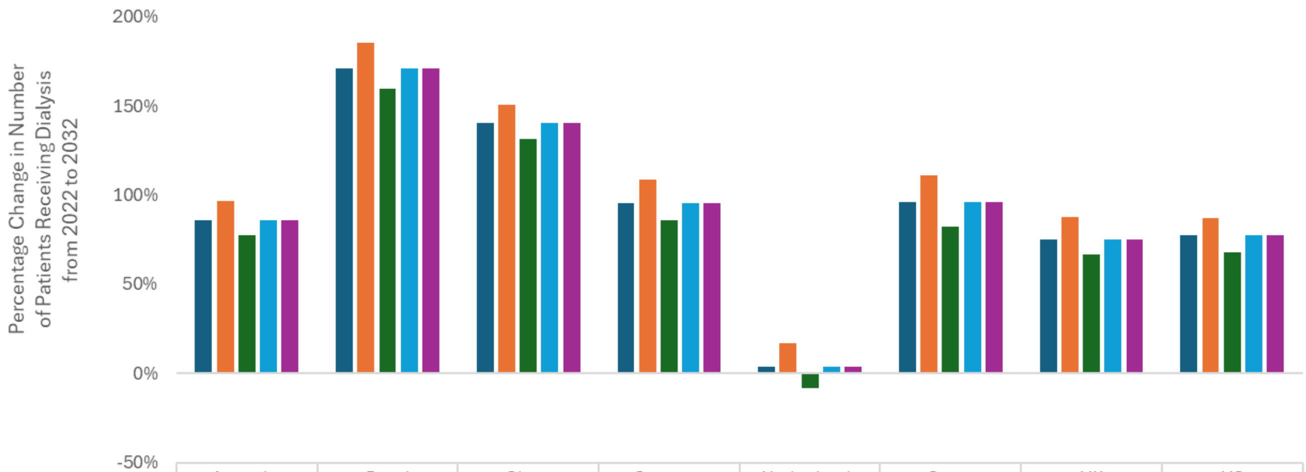

	Australia	Brazil	China	Germany	Netherlands	Spain	UK	US
Base Case	85.9%	170.8%	140.4%	95.7%	3.6%	96.3%	75.3%	77.6%
Sensitivity 1: Birth/Immigration Rate +10% and Mortality Inputs -10%	96.7%	185.4%	150.7%	108.6%	17.2%	110.8%	87.4%	87.2%
Sensitivity 2: Birth/Immigration Rate -10% and Mortality Inputs +10%	77.7%	159.5%	131.1%	86.0%	-7.9%	82.2%	66.4%	68.0%
Sensitivity 3: eGFR Decline General Population +10%	85.9%	170.8%	140.4%	95.7%	3.6%	96.3%	75.3%	77.5%
Sensitivity 4: eGFR Decline General Population -10%	85.9%	170.8%	140.4%	95.7%	3.6%	96.3%	75.3%	77.6%

Abbreviations: eGFR = estimated glomerular filtration rate; UK = United Kingdom; US = United States.

Supplementary Figure S5: Sensitivity analysis projections - percentage change in number of patients living post-transplant from 2022 to 2032

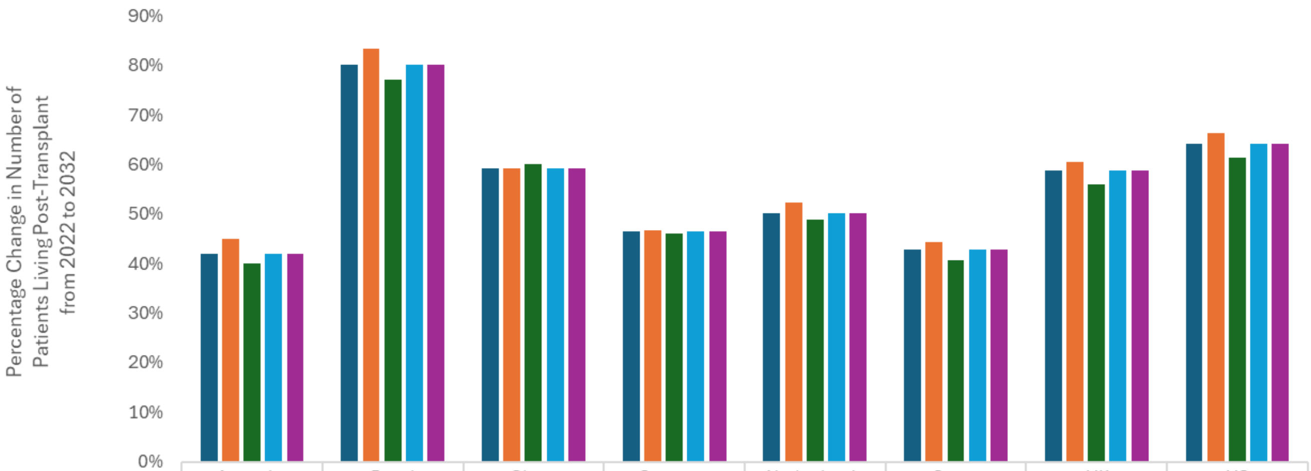

	Australia	Brazil	China	Germany	Netherlands	Spain	UK	US
Base Case	41.9%	80.1%	59.3%	46.6%	50.2%	42.8%	58.7%	64.1%
Sensitivity 1: Birth/Immigration Rate +10% and Mortality Inputs -10%	45.0%	83.5%	59.3%	46.8%	52.3%	44.3%	60.6%	66.3%
Sensitivity 2: Birth/Immigration Rate -10% and Mortality Inputs +10%	40.1%	77.1%	60.0%	46.1%	48.9%	40.6%	56.1%	61.3%
Sensitivity 3: eGFR Decline General Population +10%	41.9%	80.1%	59.3%	46.6%	50.2%	42.8%	58.7%	64.1%
Sensitivity 4: eGFR Decline General Population -10%	41.9%	80.1%	59.3%	46.6%	50.2%	42.8%	58.7%	64.1%

Abbreviations: eGFR = estimated glomerular filtration rate; UK = United Kingdom; US = United States.

Supplementary Figure S6: Ten-year lost FTEs in diagnosed CKD patients and caregivers

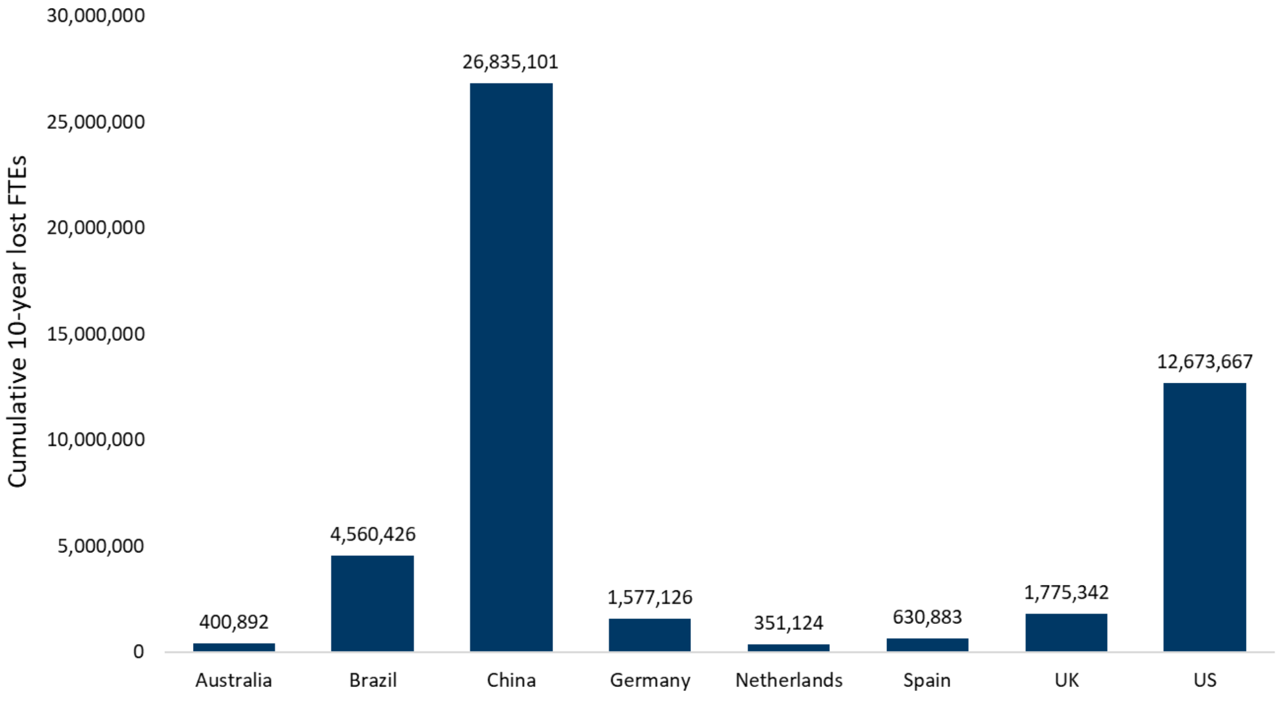

Abbreviations: CKD = chronic kidney disease; FTE = full-time equivalent; UK = United Kingdom; US = United States.

Supplementary Figure S7: Ten-year lost GDP in diagnosed CKD patients and caregivers

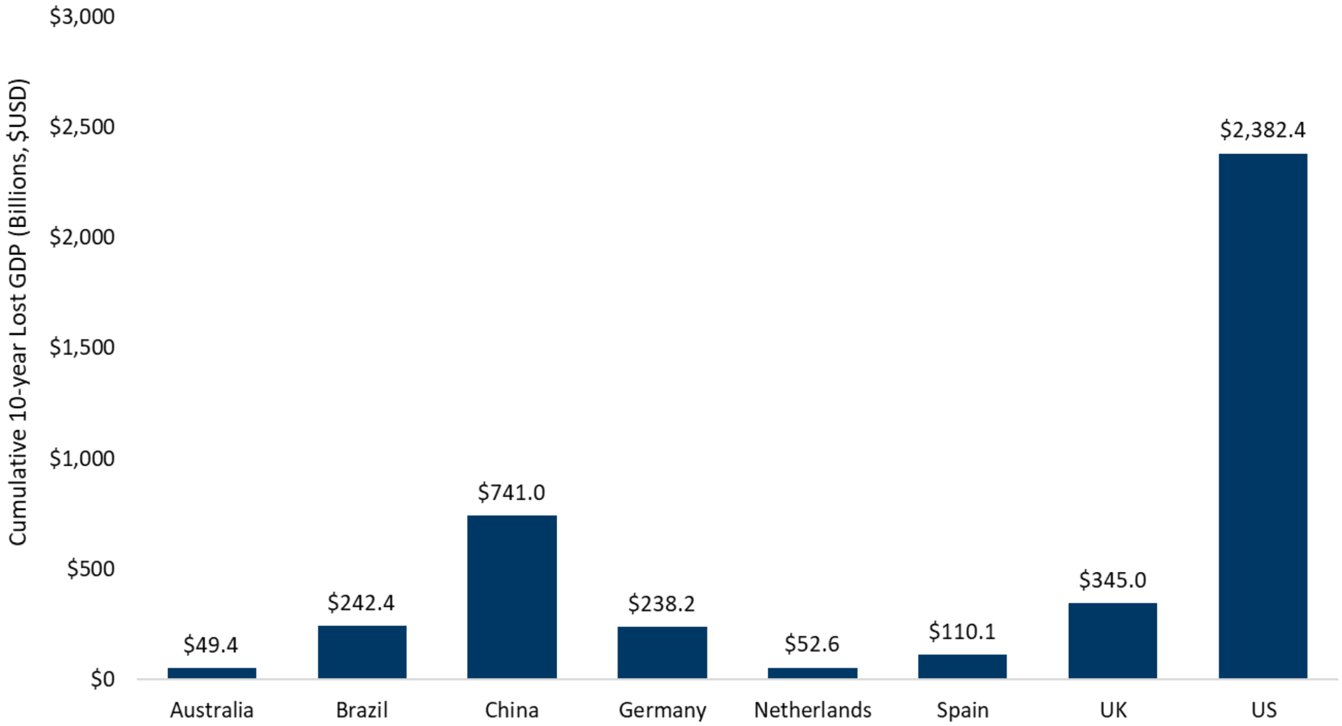

Abbreviations: CKD = chronic kidney disease; GDP = gross domestic product; UK = United Kingdom; US = United States.

Supplementary Figure S8: Ten-year lost tax revenue due to diagnosed CKD patients and caregiver absenteeism

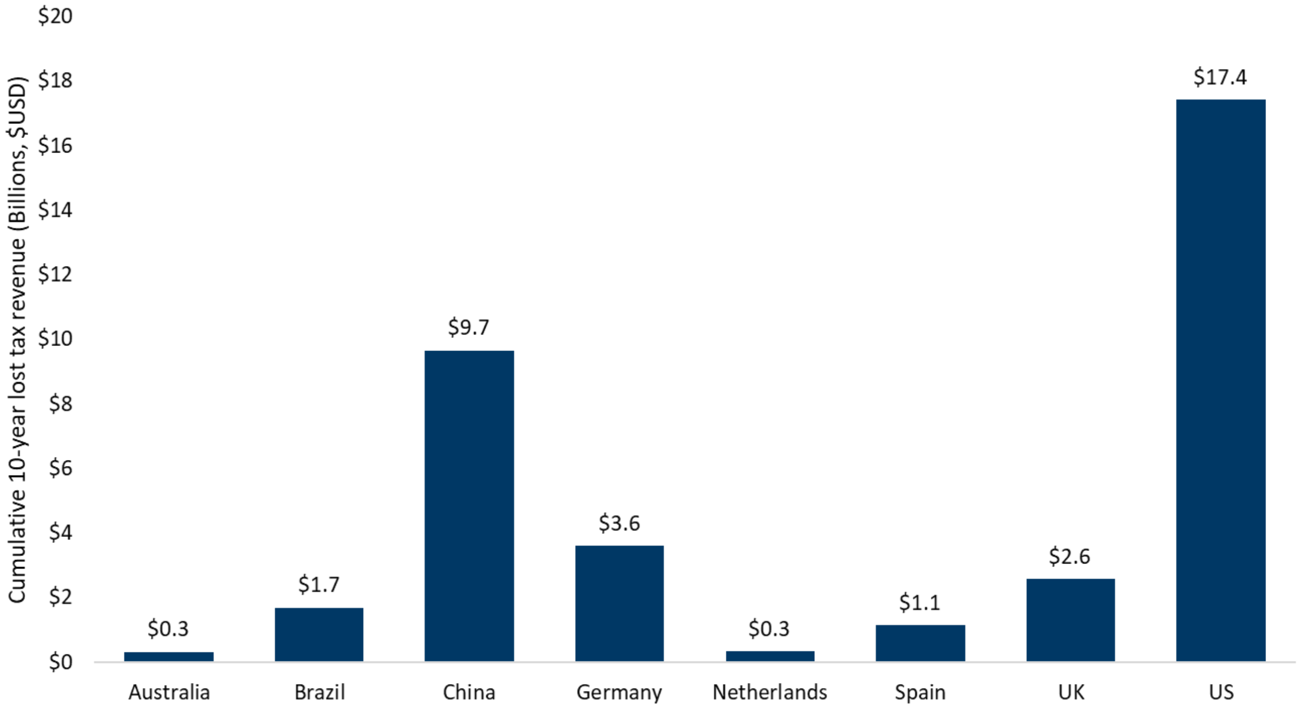

Abbreviations: CKD = chronic kidney disease; UK = United Kingdom; US = United States; USD = United States dollar.

Supplementary Tables

Supplementary Table S1a: Model values and validation targets by country (Australia, Brazil, China, Germany)

	Australia		Brazil		China		Germany	
**Parameter**	**Validation Target**	**Model Output**	**Validation Target**	**Model Output**	**Validation Target**	**Model Output**	**Validation Target**	**Model Output**
Baseline characteristics								
% Female in Total Population	50.5%[S1]	50.38%	50.82%[S2]	50.97%	48.87%[S3]	48.94%	50.77%[S4]	50.66%
Average age of Total Population (years) [SD]	38.4[S1]	39.32 [23.34]	35.36[S2]	35.44 [21.60]	37.9[S5]	38.54 [21.60]	47.8[S6]	44.55 [24.52]
CKD stage distribution at baseline								
Proportion of CKD Stage 1-2	63.7%[S7]	67.5%	54.4%[S8]	54.1%	73.3%[S9]	69.0%	66.0%[S10]	61.4%
Proportion of CKD Stage 3-5	36.3%[S7]	32.5%	45.6%[S8]	45.9%	26.7%[S11]	31.0%	34.0%[S10]	38.6%
KRT at baseline								
Prevalence of transplants at baseline (per million)	505[S12]	511	280[a]	297	141[a]	135	569[S13]	577
Prevalence of dialysis at baseline (per million)	536[S12]	575	696[S14]	637	434[S15]	540	1,186[S16,S17]	1,116
AKI								
Average AKI events/year in population (CKD and non-CKD) (per million) [SD]	5,080-16,000[S18]	15,513 [299]	5,300-20,700[S19]	11,930 [1909]	20,300[S20]	15,631 [1538]	5,300-20,700[S19]	19,502 [1130]
CV Events								
Average Stroke events/year in population (per million) [SD]	1,540[S21]	1,739 [282]	1,477[S22]	1,503 [205]	2,788[S23]	2,831 [389]	1,055[S24]	1,088 [87]
Average MIs/year in population (per million) [SD]	3,390[S25]	1,570 [180]	1,080[S26]	1,128 [152]	755[S27]	782 [109]	2,628[S28]	2,616 [166]

Mortality								
Mortality (%) in population (CKD and non-CKD) over 10 years	0.51-0.73[S29,S30]	0.71	0.65[S31]	0.61	0.79[S32]	0.78	1.24[S4]	1.21

[a]Clinical Expert Opinion

Notes: Validation targets may have been converted using country population sizes in 2022 to facilitate comparison with model outputs. The SD for AKI, stroke, and MI are calculated across the 10-year model time horizon; therefore, large SD are expected, as the population ages and more patients develop CKD, and in turn more patients experience clinical events.

Abbreviations: AKI = acute kidney injury; CKD = chronic kidney disease; CV = cardiovascular; KRT = kidney replacement therapy; MI = myocardial infarction; SD = standard deviation.

Supplementary Table S1b: Model values and validation targets by country (Netherlands, Spain, UK, US)

	Netherlands		Spain		UK		US	
**Parameter**	**Validation Target**	**Model Output**	**Validation Target**	**Model Output**	**Validation Target**	**Model Output**	**Validation Target**	**Model Output**
Baseline characteristics								
% Female in Total Population	50.28[S33]	50.28	51.04%[S34]	50.99	50.60[S35]	50.59	50.5[S36]	50.96
Average age of Total Population (years) [SD]	42.4[S37]	41.96 [23.53]	44.09[S38]	43.59 [23.03]	40.30[S35]	40.61 [23.96]	38.1[S36]	39.55 [23.87]
CKD stage distribution at baseline								
Proportion of CKD Stage 1-2	60.1%[S39]	58.4%	66.0%[S40]	64.7%	60.1%[S41]	57.2%	54.4%[S8]	59.8%
Proportion of CKD Stage 3-5	39.9%[S39]	41.6%	34.0%[S40]	35.3%	39.9%[S41]	42.8%	45.6%[S8]	40.2%
KRT at baseline								
Prevalence of transplants at baseline (per million)	677.9[S42]	705	743.8[S42]	801	581[S43]	601	733[S44]	833
Prevalence of dialysis at baseline (per million)	365.3[S45]	390	618.1[S42]	650	438[S46]	494	1,734[S8]	1,871
AKI								
Average AKI events/year in population (CKD and non-CKD) (per million) [SD]	5,300-20,700[S19]	18,029 [2566]	13,730[S47]	18,513 [2144]	5,300- 20,700[S19]	16,498 [1628]	12,500[S48]	14,955 [2251]

	Netherlands		Spain		UK		US	
Parameter	Validation Target	Model Output	Validation Target	Model Output	Validation Target	Model Output	Validation Target	Model Output
CV Events								
Average Stroke events/year in population (per million) [SD]	496[S49]	499 [57]	1,873-2,520[S50]	1,986 [212]	1,040-1,500[S51,S52]	1,067 [94]	1,871[S53]	1,818 [223]
Average MIs/year in population (per million) [SD]	3,800[S54]	3,634 [382]	706-1,975[S55]	1,510 [164]	1,341[S56]	1,580 [133]	1,856[S57]	1,920 [241]
Mortality								
Mortality (%) in population (CKD and non-CKD) over 10 years	0.62-0.98[S58]	0.84	0.82%[S59]	0.88%	1.04% [S35]	1.06%	0.87%[S60]	0.79%

Notes: Validation targets may have been converted using country population sizes in 2022 to facilitate comparison with model outputs. Due to lack of data, validation targets for some countries were assumed similar to UK. The SD for AKI, stroke, and MI are calculated across the 10-year model time horizon; therefore, large SD are expected, as the population ages and more patients develop CKD, and in turn more patients experience clinical events.

Abbreviations: AKI = acute kidney injury; CKD = chronic kidney disease; CV = cardiovascular; KRT = kidney replacement therapy; MI = myocardial infarction; SD = standard deviation.

Supplementary Table S2: Summary of data inputs by country

	**Source**							
**Data Inputs**	**Australia**	**Brazil**	**China**	**Germany**	**Netherlands**	**Spain**	**UK**	**US**
**Clinical Inputs**								
Population by age and sex	AIHW, 2022[S61]	US Census Bureau, 2022[S62]	United Nations, 2019[S63]	Destatis - Statistisches Bundesamt, 2022[S4]	Netherlands Statline, 2022[S33]	INE, 2022[S34]	UK ONS, 2021[S64]	US Census Bureau, 2022.[S65]
eGFR decline	DISCOVER CKD[27,S66]							
Diabetes prevalence	ABS, 2021[S67]	Malta et al., 2019[S68]	Li et al., 2020[S69]	Heidemann et al., 2013[S70]; Tamayo et al., 2016[S71]	Netherlands StatLine, 2022[S33]	Soriguer et al., 2012[S72]	HSE 2019, NHS Digital[S73]	CDC, 2020[S74]
Hypertension prevalence	AIHW, 2019[S75]	Malta et al., 2016[S76]	Zhang et al., 2023[S77]	Neuhauser et al., 2013[S80]	Netherlands StatLine, 2022[S33]	Corbatón-Anchuelo et al., 2018[S79]	HSE 2019, NHS Digital[S73]	NCHS, 2017-2018[S80]
HF prevalence	Liew et al., 2020[S81]	Oliveira et al., 2020[S82]	Sun et al., 2009[S83]	Störk et al., 2017[S84]	Dutch Heart Foundation, 2021[S85]	Farré et al., 2017[S86]	BHF, 2020[S56]	AHA, 2022[S87]
MI history	ABS, 2018[S88]	AHA, 2022[S88]	Li et al., 2020[S89]	Gößwald et al., 2013[S90]	Dutch Heart Foundation, 2021[S85]	de Miguel-Yanes et al., 2021[S55]	BHF, 2020[S56]	AHA, 2022[S87]
Stroke history	AIHW, 2023[S91]	Bensenor et al., 2015[S92]	IHME, 2019[S22]	Busch et al., 2013[S93]	Dutch Heart Foundation, 2021[S85]	Boix et al., 2006[S94]	BHF, 2020[S57], HSE, NHS Digital, 2012[S95]	AHA, 2022[S87]
Prevalence of KRT in population	ANZDATA Registry, 2022[S96]	Luxardo et al., 2022[S97]	UN World Population Prospects data, 2019[S98]; Zhang et al., 2020[S99]	Frei & Schober-Halstenberg, 2005[S100]	ERA-EDTA Registry, 2019[S42]	Escobar et al., 2016[S101]; REER, 2020[S102]	UK Renal Registry Report, 2022.[S103]	USRDS, 2022[S104]
eGFR distribution by age and sex	AHS, 2013[S105]	Piccolli et al., 2017[S106]	Zhang et al., 2012[S107]	Girndt et al., 2016[S108]	HSE 2016[S109]	Otero et al., 2010[S110]	HSE 2016[S109]	NHANES, 2017-2020[S111]
Albuminuria distribution by age and sex	AHS, 2013[S105]	Piccolli et al., 2017[S106]	Japanese Society of Nephrology, 2012.[S112]	Girndt et al., 2016[S108]	HSE 2016[S109]	Gorostidi et al., 2018[S113]	HSE 2016[S109]	NHANES 2017-2020[S111]

	Source							
**Data Inputs**	**Australia**	**Brazil**	**China**	**Germany**	**Netherlands**	**Spain**	**UK**	**US**
Diagnosed CKD by stage	Hirst et al., 2020[5]							
Diabetes prevalence in CKD	USRDS 2020[S114]	USRDS 2020[S114]	USRDS 2020[S114]	USRDS 2020[S114]	USRDS 2020[S114]	Escobar et al., 2021[S115]	USRDS, 2022[S116]	USRDS, 2020[S114]
Hypertension prevalence in CKD	USRDS 2010[S117]	USRDS 2010[S117]	USRDS 2010[S117]	Schneider et al., 2018[S118]	USRDS 2010[S117]	Escobar et al., 2021[S115]	USRDS, 2010[S119]	USRDS, 2010[S117]
HF prevalence in CKD	Estimated[a]	USRDS 2018[S120]	Yuan et al., 2017[S121]	Beck et al., 2015[S122]	USRDS 2018[S120]	Escobar et al., 2021[S115]	USRDS 2018[S122]	USRDS, 2018[S120]
MI history in CKD	USRDS 2022[S104]	USRDS 2022[S104]	Yuan et al., 2017[S121]	USRDS 2022[S104]	USRDS 2022[S104]	Escobar et al., 2021[S115]	USRDS 2022[S116]	USRDS, 2022[S104]
Stroke history in CKD	Estimated[b]	USRDS 2022[S104]	Masson et al., 2015[S124]	USRDS 2022[S104]	USRDS 2022[S104]	Escobar et al., 2021[S115]	USRDS 2022[S116]	USRDS, 2022[S104]
Incidence of diabetes	AIHW, 2020[S125]	CDC, 2019[S126]	Wang et al., 2020[S127]	National Diabetes Surveillance, 2019[S128]	Ubink-Veltmaat et al., 2003[S129]	Rojo-Martínez et al., 2020[S130]	Zghebi et al., 2017[S131]	CDC, 2019[S126]
Incidence of hypertension	Dannenberg 1988[S132]	Dannenberg 1988[S132]	Liang et al., 2015[S133]	Dannenberg 1988[S132]	Dannenberg 1988[S132]	Beunza et al., 2006[S134]	Dannenberg 1988.[S132]	Dannenberg 1988.[S132]
Incidence of HF	Liew et al., 2020[S81]	Oliveira et al., 2020[S82]	Wang et al., 2021[S135]	Störk et al., 2017[S854]	Dutch Heart Foundation, 2021[S85]	Fernández-Gassó et al., 2019[S136]	BHF, 2020[S56]	Khera, 2017[S137]
Incidence of MI	Nedkoff et al., 2011[S138]	Mefford, 2020[S139]	Kaptoge et al., 2019[S140]	Krämer et al., 2021[S141]	Dutch Heart Foundation, 2021[S85]	Forcadell et al., 2018[S142]	BHF, 2020[S56]	Mefford, 2020[S139]
Incidence of stroke	AIHW, 2023[S91]	de Santana et al., 2018[S143]	AstraZeneca Data on File, 2022[S98]	Stahmeyer et al., 2019[S144]	Dutch Heart Foundation, 2021[S85]	Vega et al., 2009[S145]	BHF, 2020[S56]	Madsen, 2020[S146]
Incidence of HHF	AIHW, 2023[S91]	NCHS Data Brief, 2012[S147]	Tseng 2011[S148]	Lawson et al., 2019[S149]	Meems et al., 2020[S150]	Anguita Sánchez et al., 2021[S151]	Lawson et al., 2019[S149]	NCHS Data Brief, 2012[S147]
Relative risk of diabetes in CKD	Lin et al., 2019[S152]	Lin et al., 2019[S152]	Lin et al., 2019[S152]	Lin et al., 2019[S152]	Lin et al., 2019[S152]	Lin et al., 2019[S152]	Lin 2019[S153]	Zelnick, 2017[S154]

	Source							
**Data Inputs**	**Australia**	**Brazil**	**China**	**Germany**	**Netherlands**	**Spain**	**UK**	**US**
Relative risk of hypertension in CKD	Hounkpatin et al., 2020[S155]	Hounkpatin et al., 2020[S155]	Hounkpatin et al., 2020[S155]	Hounkpatin et al., 2020[S155]	Hounkpatin et al., 2020[S155]	Hounkpatin et al., 2020[S155]	Hounkpatin 2020[S155]	AHA, 2010[S156]
Relative risk of HF in CKD	USRDS, 2009[S157]							
Relative risk of MI in CKD	USRDS, 2009[S157]							
Relative risk of stroke in CKD	Masson et al., 2015[S124]							
Rate ratio of HHF in CKD	Bansal et al., 2019[S158]							
Incidence of AKI	AIHW, 2015[S159]	Sawhney et al., 2022[S160]	Liu et al., 2015[S161]	Matrisch et al., 2023[S162]	Sawhney et al., 2022[S160]	Sawhney et al., 2022[S160]	Sawhey, 2022[S160]	Sawhney, 2022[S160]
Relative risk of AKI in CKD	Grams et al., 2015[S163]							
Outcomes associated with AKI	Duarte et al., 2022[S164]	Duarte et al., 2022[S164]	Duarte et al., 2022[S164]	Duarte et al., 2022[S164]	Duarte et al., 2022[S164]	Duarte et al., 2022[S164]; Soler et al., 2023[S47]	Duarte et al., 2022[S164]	Duarte et al., 2022[S164]
eGFR eligibility threshold for transplant	The Transplantation Society of Australia and New Zealand, 2022[S165]	Hecking et al., 2022[S166]	Han et al., 2016[S167]	Expert opinion	Dutch guideline for diabetic nephropathy, 2020	Expert opinion	Expert opinion	Expert opinion
Probability of transplant	ANZDATA 2020[S168]	Lugon et al., 2018[S169]	Zhang et al., 2020[S199]	Statista, 2021[S170]	UK Renal Registry, 2022[S103]	Escobar et al., 2016[S101], Crespo et al., 2021[S171], Statista, 2019[S172]	UK Renal Registry, 2022[S103]	USRDS, 2022[S104]
Probability of transplant after dialysis	ANZDATA 2020[S168]	Cristelli et al., 2021[S13]	Chinese National Renal Data System[S173]	UK renal registry, 2022[S103]	Nefrovisie, 2022[S174]	UK Renal Registry, 2022[S103]	UK Renal Registry, 2022[S103]	USRDS, 2022[S104]

	Source							
**Data Inputs**	**Australia**	**Brazil**	**China**	**Germany**	**Netherlands**	**Spain**	**UK**	**US**
Probability of dialysis	Assumption	Hecking 2022[S166]	Liu et al., 2020[S175]	Chan et al., 2019[S176]	KDIGO 2019[S177]	UK Renal Registry 2022[S103]	UK Renal Registry 2022[S103]	KDIGO, 2019[S177]<br><br>USRDS, 2022[S104]
MI mortality rate	Camacho et al., 2022[S178]	Brant 2022[S179], Ferreira 2009[S180]	Long et al., 2020[S181]	Asaria et al., 2022[S182]	Jones et al., 2019[S183]	Perez 1998[S184]	Asaria et al., 2022[S182]	Asaria et al., 2022[S182]
Stroke mortality rate	Thrift et al., 2000[S185]	Minelli et al., 2007[S186]	He et al., 2022[S187]	Koton et al., 2014[S188]	Vaartjes et al., 2013[S189]	Diaz-Guzman 2012[S190]	BHF, 2021	CDC, 2021 [S191]
AKI mortality rate	Bendall et al., 2021[S192]	Dos Santos et al., 2019[S193], Inda-Filho et al., 2021[S194]	Think Kidneys, 2018,[S195] Yang et al. 2015,[S196] Xu et al. 2015[S197]	Khadzhynov et al., 2019[S198]	Uhel et al., 2020[S199]	Think Kidneys 2018[S195]	Think Kidneys 2018[S195]	Sohaney et al., 2022[S200]
HF mortality rate	Teng et al., 2010[S201]	Fernandes et al., 2020[S202]	Feng et al., 2022[S203]	Jones et al., 2019[S183]	Jones et al., 2019[S183]	Jones et al., 2019[S183]	Taylor 2020[S204]	Taylor 2020[S204]
Transplant morality rate	Wyld et al., 2021[S205]	Foresto et al., 2020[S206]	Zhang et al., 2022[S207]	Mehrabi et al.,2004[S208]	Renine Annual Report, 2022[S174]	Ojo et al., 2013[S209]	UK Renal Registry, 2022[S103]	USRDS, 2022[S104]
Mortality RR by CKD	Levey et al, 2011[S210]							
Mortality rates by age and sex	ABS, 2021[S211]	WHO, 2020[S212]	Zhu et al., 2019[S213]	Destatis - Statistisches Bundesamt, 2022[S214]	Netherlands Statline, 2022[S33]	INE, 2021[S215]	UK Office for National Statistics	Statista.com, 2019 [S216]
Dialysis mortality	ANZDATA, 2021[42]							
Healthcare cost inputs								
CKD cost by stage	Wyld et al., 2015[S217]	AstraZeneca 2021 (DAPA-CKD)	Wu and Yang, 2013[S218]	Gandjour et al., 2020[S219]	vanOosten et al., 2019[S220]	Pons et al., 2006[S221], Lorenzo-Sallares et al., 2014[S222]	AstraZeneca 2021 (DAPA-CKD)	Golestaneh et al., 2017[S223]

	Source							
**Data Inputs**	**Australia**	**Brazil**	**China**	**Germany**	**Netherlands**	**Spain**	**UK**	**US**
KRT cost	Cass et al., 2010[S224], NHS Improvement, 2019[S225], Howard et al., 2010[S226]	Sesso et al., 2020[35], Silva et al., 2016[S227]	Wang et al., 2019;[S228] Zhao et al., 2012;[S229] Zhang et al., 2020[S16] AstraZeneca Data on File, 2022[S98]	Shukri et al., 2022,[S230] Kleophas et al., 2007[S231]	Mohnen et al., 2019[S232]	Lorenzo-Sallares et al., 2014[S222], Ministerio de Sanidad, 2021[S233], Arrieta et al., 2011[S234]	NHS England and NHS Improvement, 2020[S225], NHS Improvement, 2019[S235], NHS Blood and Transplant, 2017[S236]	USRDS, 2020[S237], Elbasha et al., 2017[S238], Axelrod et al., 2018[S239]
Utility Input								
Utility weights by CKD stage	Cooper et al., 2020[S240]							
Societal inputs								
Absenteeism and presenteeism in CKD patients	van Haalen, 2020[S241]	van Haalen, 2020[S241]	van Haalen, 2020[S241]	van Haalen, 2020[S241]	van Haalen, 2020[S241]	van Haalen et al., 2020[S241]	van Haalen, 2020[S241]	van Haalen, 2020[S241]
Absenteeism and presenteeism in caregivers	Eriksson, 2017[S242]							
Environmental inputs								
Environmental outputs by stage	AstraZeneca Data on File[S243]	AstraZeneca Data on File[S243]	AstraZeneca Data on File[S243]	AstraZeneca Data on File[S243]	AstraZeneca Data on File[S243]	AstraZeneca Data on File[S243]	AstraZeneca Data on File[S243]; Zoccali et al. 2023[29]	AstraZeneca Data on File[S243]

[a] Calculated based on the relative risk of heart failure by CKD stage multiplied by the general population prevalence of heart failure.

[b] Calculated based on the relative risk of stroke by CKD stage multiplied by the general population prevalence of heart failure.

Abbreviations: ABS = Australian Bureau of Statistics; AHA = American Heart Association; AHS = Australian Health Survey; AIHW = Australian Institute of Health and Welfare; AKI = acute kidney injury; BHF = British Heart Foundation; CDC = Centres for Disease Control and Prevention; CKD = chronic kidney disease; CV = cardiovascular; DAPA-CKD = dapagliflozin and prevention of adverse outcomes in chronic kidney disease; HHF = hospitalization due to heart failure; HF = heart failure; HSE = Heart Survey for England; IHME = Institute for Health Metrics and Evaluation; INE = Instituto Nacional de Estadística; KDIGO = Kidney Disease Improving Global Outcomes; NCHS = National Center for Health Statistics; NHANES = National Health and Nutrition Examination Survey; NICE DSU = National Institute for Health and Care Excellence Decision Support Unit; RR = relative risk; UK = United Kingdom; US = United States; USRDS = The United States Renal Data System; WHO = World Health Organization.

Supplementary Table S3: Summary of population characteristics at baseline (2022) by country

Population Characteristic	Australia	Brazil	China	Germany	Netherlands	Spain	UK	US
Age Distribution								
Age Distribution (0-34 years)	44.8%	50.5%	44.1%	36.5%	40.7%	35.2%	42.9%	45.0%
Age Distribution (35-64 years)	38.1%	39.1%	42.8%	41.3%	39.3%	44.8%	38.4%	37.9%
Age Distribution (65+ years)	17.1%	10.4%	13.1%	22.2%	20.0%	20.0%	18.7%	17.1%
CKD Stage Distribution								
Stage distribution (CKD 1-2)	67.6%	54.1%	69.0%	61.4%	58.4%	64.7%	57.2%	59.8%
Stage distribution (CKD 3-5)	32.4%	45.9%	31.0%	38.6%	41.6%	35.3%	42.8%	40.2%
Prevalence of KRT[a]								
Prevalent dialysis (PMP)	575.0	637.0	540.0	1116.0	390.0	650.0	494.0	1871.0
Prevalent transplant (PMP)	511.0	297.0	135.0	577.0	705.0	801.0	601.0	833.0

[a] Prevalence was calculated using baseline KRT modelled at baseline (**Table** 1) and population data at baseline.

Abbreviations: KRT = kidney replacement therapy; PMP = per million population; UK = United Kingdom; US = United States.

Supplementary Table S4: Predicted freshwater consumption, fossil fuel depletion, and overall carbon footprint from KRT patients in 2022 and 2032

Outcome	Year	Australia	Brazil	China	Germany	Netherlands	Spain	UK	US
Freshwater Consumption (m^3)	2022	1.06 M	22.83 M	35.87 M	5.32 M	570.52 K	1.97 M	1.70 M	153.13 M
	2032	1.96 M	60.74 M	85.45 M	10.34 M	638.10 K	3.79 M	3.00 M	274.41 M
	2022 to 2032	84.6%	166.0%	138.3%	94.3%	11.8%	92.6%	76.6%	79.2%
Fossil Fuel Depletion (kg oil eq.)	2022	181.57 M	319.98 M	2.26 B	233.15 M	19.69 M	90.78 M	89.33 M	2.14 B
	2032	332.23 M	850.24 M	5.36 B	449.66 M	23.23 M	171.17 M	156.20 M	3.82 B
	2022 to 2032	83.0%	165.7%	136.8%	92.9%	18.0%	88.6%	74.9%	78.4%
Overall Carbon Footprint (kg CO_2 eq.)	2022	60.15 M	939.05 M	6.40 B	642.08 M	54.00 M	215.24 M	200.80 M	5.16 B
	2032	109.96 M	2.50 B	15.16 B	1.24 B	63.20 M	407.41 M	351.59 M	9.23 B
	2022 to 2032	82.8%	166.2%	137.0%	93.1%	17.1%	89.3%	75.1%	78.7%

Abbreviations: B = billion; CO_2 = carbon dioxide; eq = equivalent; K = thousand; kg = kilogram; M = million; UK = United Kingdom; US = United States.

Supplementary Table S5: Growth rate of incident dialysis from 2022 to 2032

	Australia	Brazil	China	Germany	Netherlands	Spain	UK	US
Growth rate of incident dialysis	3.25%	4.8%	5.6%	4.74%	0.25%	3.36%	2.0%	1.6%

Abbreviations: UK = United Kingdom; US = United States.

Supplementary Table S6: Percentage of CKD patients and the number of diagnosed patients in CKD stages 3 and 4, annual mortality rate in stage 4, and yearly stage 4 cost input

	Year	Australia	Brazil	China	Germany	Netherlands	Spain	UK	US
Percentage of CKD Patients in Stage 3	2022	28.94%	41.46%	29.47%	32.65%	39.54%	31.33%	39.60%	34.62%
	2032	31.77%	37.38%	30.47%	41.84%	44.86%	40.85%	42.20%	34.99%
Number of Diagnosed Stage 3 Patients	2022	392,801	5,469,391	23,639,880	1,850,301	444,077	990,977	1,667,228	8,885,824
	2032	554,261	5,769,448	30,076,361	2,470,917	638,242	1,607,450	2,026,214	10,389,573
	2022 to 2032	↑41.1%	↑5.5%	↑27.2%	↑33.5%	↑43.7%	↑62.2%	↑21.5%	↑16.9%
Percentage of CKD Patients in Stage 4	2022	1.29%	1.92%	0.53%	2.47%	0.62%	1.50%	1.20%	2.03%
	2032	3.33%	5.06%	2.44%	3.68%	4.35%	4.15%	3.71%	5.14%
Number of Diagnosed Stage 4 Patients	2022	27,408	389,216	673,021	219,011	10,748	73,620	79,730	811,568
	2032	98,564	1,276,527	4,075,202	372,353	105,491	275,838	304,515	2,499,943
	2022 to 2032	↑259.6%	↑228.0%	↑505.5%	↑70.0%	↑881.5%	↑274.7%	↑281.9%	↑208.0%
Annual Mortality Rate in Stage 4 Patients	2022	3.17%	1.72%	2.77%	8.98%	1.81%	5.17%	5.56%	1.78%
Yearly Stage 4 Cost Input	All	$17,310	R$4,850	¥42,824	€4,976	€12,286	€3,836	£1,252	$16,725

Abbreviations: CKD = chronic kidney disease; UK = United Kingdom; US = United States; ↑ = increase.

Supplementary References

S1. Statistics ABo. Regional population by age and sex. Updated 30/08/2022. https://www.abs.gov.au/statistics/people/population/regional-population-age-and-sex/latest-release#key-statistics

S2. (IDB) ID. Population estimates and projections for 227 countries and areas. https://www.census.gov/data-tools/demo/idb/#/country?COUNTRY_YEAR=2023&COUNTRY_YR_ANIM=2023&FIPS_SINGLE=BR

S3. Statista. Population in China from 2012 to 2022, by gender. Updated Jan 17, 2023. https://www.statista.com/statistics/251129/population-in-china-by-gender/#:~:text=In%202022%2C%20there%20were%20around,1.41%20billion%20people%20in%20total

S4. Destatis - Statistisches Bundesamt. 12411-006 Population: Germany, reference date, age, nationality/sex/marital status. https://www-genesis.destatis.de/genesis//online?operation=table&code=12411-0006&bypass=true&levelindex=0&levelid=1678288674953#abreadcrumb

S5. Economics W. China's Median Age. https://www.worldeconomics.com/Demographics/Median-Age/China.aspx#:~:text=It%20is%20a%20single%20index,global%20value%20of%2030.3%20years

S6. World Data. Median age by country. Updated 2021. https://www.worlddata.info/average-age.php

S7. Welfare AIoHa. *Australian Institute of Health and Welfare - CKD Data Tables*. 2013. https://www.aihw.gov.au/reports/chronic-kidney-disease/chronic-kidney-disease/data

S8. United States Renal Data System. *2022 USRDS Annual Data Report: CKD in the General Population*. 2022. Accessed 2023. https://usrds-adr.niddk.nih.gov/2022/chronic-kidney-disease/1-ckd-in-the-general-population

S9. Wang F, He K, Wang J, et al. Prevalence and Risk Factors for CKD: A Comparison Between the Adult Populations in China and the United States. *Kidney Int Rep*. Sep 2018;3(5):1135-1143. doi:10.1016/j.ekir.2018.05.011

S10. Bruck K, Stel VS, Gambaro G, et al. CKD Prevalence Varies across the European General Population. *J Am Soc Nephrol*. Jul 2016;27(7):2135-47. doi:10.1681/ASN.2015050542

S11. Zhuang Z, Tong M, Clarke R, Wang B, Huang T, Li L. Probability of chronic kidney disease and associated risk factors in Chinese adults: a cross-sectional study of 9 million Chinese adults in the Meinian Onehealth screening survey. *Clinical Kidney Journal*. 2022;15(12):2228-2236.

S12. Australia & New Zealand Dialysis & Transplant Registry. *ANZDATA 44th Annual Report 2021 (Data to 2020)*. 2021. Accessed 2023. https://www.anzdata.org.au/report/anzdata-44th-annual-report-2021-data-to-2020/

S13. Frei U, Schober-Halstenberg H-J. Nierenersatztherapie in Deutschland - Bericht über Dialysebehandlung und Nierentransplantation in Deutschland (2006-2007). QuaSi-Niere; 2008. Accessed April 25, 2023 https://www.bundesverband-niere.de/wp-content/uploads/2019/02/QuaSi-Niere-Bericht_2006-2007.pdf

S14. Nerbass FB, Lima HdN, Thomé FS, Vieira Neto OM, Lugon JR, Sesso R. Brazilian dialysis survey 2020. *Brazilian Journal of Nephrology*. 2022;44:349-357.

S15. Zhang L, Zhao MH, Zuo L, et al. China Kidney Disease Network (CK-NET) 2016 Annual Data Report. *Kidney Int Suppl (2011)*. Dec 2020;10(2):e97-e185. doi:10.1016/j.kisu.2020.09.001

S16. Medical Netcare GmbH (MNC). Management im Gesundheitswesen. “Jahresberichte zur Qualitätssicherung in der Dialyse”. 2019. Accessed April 25, 2023. https://m-nc.de/qs-dialyse-jahresberichte. S17. Federal Statistical Office - Federal Health Monitoring, and Statistisches Bundesamt - Gesundheitsberichterstattung des Bundes. “Treatment procedures for dialysis patients (number)”. Gesundheit Statistik Gesundheitsberichterstattung Des Bundes; 2017. Accessed April 25, 2023. https://www.gbe-bund.de/gbe/!pkg_olap_tables.prc_set_page?p_uid=gast&p_aid=91089978&p_sprache=D&p_help=2&p_indnr=878&p_ansnr=39438360&p_version=7&D.000=3732

S18. Australian Institute of Health and Welfare. *Acute kidney injury in Australia: A first national snapshot*. 2015. Accessed 2023. https://www.aihw.gov.au/getmedia/7e0f5313-d61d-4de3-ad8d-389dcc7a03dc/19380.pdf.aspx?inline=true

S19. Acute kidney injury (AKI) in England – a report on the nationwide collection of AKI warning test scores from 2018 (2020).

S20. Cheng X, Wu B, Liu Y, Mao H, Xing C. Incidence and diagnosis of Acute kidney injury in hospitalized adult patients: a retrospective observational study in a tertiary teaching Hospital in Southeast China. *BMC Nephrol*. Jun 24 2017;18(1):203. doi:10.1186/s12882-017-0622-6

S21. Australian Institute for Health and Welfare. *Heart, stroke and vascular disease: Australian facts*. 2023. Accessed 2023. https://www.aihw.gov.au/reports/heart-stroke-vascular-diseases/hsvd-facts/contents/about

S22. Institute for Health Metrics and Evaluation. *GBD Compare*. 2015. 2023. http://vizhub.healthdata.org/gbd-compare

S23. The Writing Committee of the Report on Cardiovascular Health and Diseases in China, Hu SS. Report on cardiovascular health and diseases in China 2021: an updated summary. *J Geriatr Cardiol*. Jun 28 2023;20(6):399-430. doi:10.26599/1671-5411.2023.06.001
S24. Stroke Alliance for Europe. *The Burden of Stroke in Germany*. 2017. Accessed 2023. https://www.safestroke.eu/wp-content/uploads/2017/12/SAFE_STROKE_GERMANY.pdf
S25. Australian Institute for Health and Welfare. *Australia's health 2018: Incidence of heart attacks*. 2018. Accessed 2023. https://www.aihw.gov.au/reports/australias-health/australias-health-2018/contents/indicators-of-australias-health/heart-attacks
S26. Alves L, Polanczyk CA. Hospitalization for acute myocardial infarction: a population-based registry. *Arquivos Brasileiros de Cardiologia*. 2020;115:916-924.
S27. Ma L-Y, Chen W-W, Gao R-L, et al. China cardiovascular diseases report 2018: an updated summary. *Journal of geriatric cardiology: JGC*. 2020;17(1):1.
S28. Nimptsch U, Busse R, Mockel M, et al. Recording early deaths following emergency department visits in inpatient data: An observational study using data of 16 German hospitals. *Z Evid Fortbild Qual Gesundhwes*. Apr 2023;177:35-40. doi:10.1016/j.zefq.2022.12.003
S29. Australian Bureau of Statistics. *Deaths, Australia*. 2021. Accessed 2023. https://www.abs.gov.au/statistics/people/population/deaths-australia/
S30. Australian Bureau of Statistics. *Provisional Mortality Statistics*. 2022. Accessed 2023. https://www.abs.gov.au/statistics/health/causes-death/provisional-mortality-statistics/latest-release
S31. United Nations Depatyment of Economic and Social Affairs Population Division. *World Mortality 2019: Data Booklet*. 2019. https://www.un.org/en/development/desa/population/publications/pdf/mortality/WMR2019/WorldMortality2019DataBooklet.pdf
S32. United States Census Bureau. International Database. Accessed 2023. https://www.census.gov/data-tools/demo/idb/#/dashboard?COUNTRY_YEAR=2023&COUNTRY_YR_ANIM=2023
S33. StatLine N. Population; sex, age, generation and migration background, 1 Jan; 1996-2022. Accessed April 2023, https://opendata.cbs.nl/statline/#/CBS/en/dataset/37325eng/table?ts=1679662511855
S34. Estadistica INd. Population (Spanish/foreign) by age (year by year) and sex. Accessed April 2023, https://www.ine.es/jaxi/tabla.do?path=/t20/e245/p04/provi/l1/&file=00000002.px&type=pcaxis&L=1
S35. Office for National Statistics. *Office for National Statistics – National life tables – life expectancy in the UK: 2018 to 2020*. 2021. Accessed February 1, 2022. https://www.ons.gov.uk/peoplepopulationandcommunity/birthsdeathsandmarriages/lifeexpectancies/bulletins/nationallifetablesunitedkingdom/2018to2020#:~:text=In%20the%20UK%20the%20median,estimates%20for%202015%20to%202017.
S36. US Census Bureau. United States: Population by Age and Sex. Updated 2022. Accessed 2023, https://www.census.gov/popclock/data_tables.php?component=pyramid
S37. Statistics Netherlands. Age distribution. Updated 2022. https://www.cbs.nl/en-gb/visualisations/dashboard-population/age/age-distribution
S38. Instituto Nacional de Estadística. Average age by Autonomous Community and province, sex and size of municipality. Updated January 2022. Accessed 2023, https://www.ine.es/jaxi/Tabla.htm?path=/t20/e245/p04/provi/l1/&file=0tamu005.px&L=1
S39. Sundström J, Bodegard J, Bollmann A, et al. Prevalence, outcomes, and cost of chronic kidney disease in a contemporary population of 2· 4 million patients from 11 countries: The CaReMe CKD study. *The Lancet Regional Health–Europe*. 2022;20
S40. Gorostidi M, Sanchez-Martinez M, Ruilope LM, et al. Chronic kidney disease in Spain: Prevalence and impact of accumulation of cardiovascular risk factors. *Nefrologia (Engl Ed)*. Nov-Dec 2018;38(6):606-615. Prevalencia de enfermedad renal cronica en Espana: impacto de la acumulacion de factores de riesgo cardiovascular. doi:10.1016/j.nefro.2018.04.004
S41. NHS Digital. Health Survey for England, 2016. December 2017. Accessed September 2022. https://digital.nhs.uk/data-and-information/publications/statistical/health-survey-for-england/health-survey-for-england-2016#highlights
S42. Registry E-E. ERA-EDTA Annual Report. 2019. https://www.era-online.org/wp-content/uploads/2022/11/ERA-Registry-Annual-Report-2019.pdf
S43. Annual Report on Kidney Transplantation (2022).

S44. Organ Procurement & Transplantation Network. Transplants by Donor Type: U.S. Transplants Performed: January 1, 1988 - October 31, 2023 For Organ = Kidney. Accessed 2023, https://optn.transplant.hrsa.gov/data/view-data-reports/national-data/#
S45. Astley ME, Boenink R, Abd ElHafeez S, et al. The ERA registry annual report 2020: a summary. *Clinical kidney journal*. 2023;16(8):1330-1354.
S46. UK Renal Registry. *UK Renal Registry 24th Annual Report– data to 31/12/2020*. 2022. Accessed October 2022. https://ukkidney.org/sites/renal.org/files/publication/file-attachments/24th_UKRR_ANNUAL_REPORT_BOOK%20version%203.pdf
S47. Soler MJ, de Francisco ALM, Ramos N. Global Perspectives in Acute Kidney Injury: Spain. *Kidney360*. 2023;4(3):393-397.
S48. Rewa O, Bagshaw SM. Acute kidney injury—epidemiology, outcomes and economics. *Nature reviews nephrology*. 2014;10(4):193-207.
S49. Safe Stroke. *The Burden of Stroke in the Netherlands*. 2017. https://www.safestroke.eu/wp-content/uploads/2017/12/SAFE_STROKE_NETHERLANDS.pdf
S50. de Leciñana MA, Morales A, Martínez-Zabaleta M, Ayo-Martín Ó, Lizán L, Castellanos M. Characteristics of stroke units and stroke teams in Spain in 2018. Pre2Ictus project. *Neurología (English Edition)*. 2023;38(3):173-180.
S51. Lee S, Shafe AC, Cowie MR. UK stroke incidence, mortality and cardiovascular risk management 1999-2008: time-trend analysis from the General Practice Research Database. *BMJ Open*. Jan 1 2011;1(2):e000269. doi:10.1136/bmjopen-2011-000269
S52. Stroke Association. *Stroke Statistics* 2023. Accessed August 2023. https://www.stroke.org.uk/what-is-stroke/stroke-statistics
S53. Centers for Disease Control and Prevention NCfHS. Stroke Facts. 2022;
S54. Koopman C, Bots ML, van Oeffelen AA, et al. Population trends and inequalities in incidence and short-term outcome of acute myocardial infarction between 1998 and 2007. *International journal of cardiology*. 2013;168(2):993-998.
S55. de Miguel-Yanes JM, Jiménez-García R, Hernandez-Barrera V, et al. Sex differences in the incidence and outcomes of acute myocardial infarction in Spain, 2016–2018: a matched-pair analysis. *Journal of Clinical Medicine*. 2021;10(8):1795.
S56. British Heart Foundation. *Heart & Circulatory Disease Statistics 2020*. 2020. April 2020. Accessed October 16, 2022. https://www.bhf.org.uk/what-we-do/our-research/heart-statistics/heart-statistics-publications/cardiovascular-disease-statistics-2020S57. Centers for Disease Control and Prevention NCfHS. Heart Disease Facts. https://www.cdc.gov/heartdisease/facts.htm#:~:text=Every%20year%2C%20about%20805%2C000%20people,States%20have%20a%20heart%20attack.&text=Of%20these%2C,are%20a%20first%20heart%20attack&text=200%2C000%20happen%20to%20people%20who%20have%20already%20had%20a%20heart%20attack
S58. StatLine N. Mortality; key figures. 2022. https://opendata.cbs.nl/statline/#/CBS/en/dataset/37979eng/table?ts=1712588475379
S59. Regidor E, Mateo A, Barrio G, Fuente Ldl. Mortality in Spain in the context of the economic crisis and austerity policies. *American journal of public health*. 2019;109(7):1043-1049.
S60. Bank TW. Death rate, crude (per 1,000 people) - United States. https://data.worldbank.org/indicator/SP.DYN.CDRT.IN?locations=US
S61. Welfare AIoHa. Population: Australian population - age and sex. Updated September 2022. https://www.housingdata.gov.au/visualisation/population/australian-population-age-and-sex
S62. Bureau USC. Brazil: Population by Age and Sex. https://www.census.gov/popclock/world/br
S63. United Nations. World Population Prospects, China 2019. Accessed 2021, https://population.un.org/wpp/Download/Standard/Population/
S64. Office for National Statistics NRoS, Northern Ireland Statistics and Research Agency,. *Mid-Year Population Estimates, UK, June 2020*. 2021. Accessed February 1, 2022. https://www.ons.gov.uk/peoplepopulationandcommunity/populationandmigration/populationestimates/bulletins/annualmidyearpopulationestimates/mid2020#age-structure-of-the-uk-population
S65. Bureau U. Census."Census. gov.". *US Census Bureau*.
S66. AstraZeneca Data on File. eGFR_mulitvariate_linear_HL1. 2022.
S67. Statistics ABo. Diabetes prevalence. Updated 2021. https://www.abs.gov.au/statistics/health/health-conditions-and-risks/diabetes/latest-release

S68. Malta DC, Duncan BB, Schmidt MI, et al. Prevalence of diabetes mellitus as determined by glycated hemoglobin in the Brazilian adult population, National Health Survey. *Revista Brasileira De Epidemiologia*. 2019;22
S69. Li Y, Teng D, Shi X, et al. Prevalence of diabetes recorded in mainland China using 2018 diagnostic criteria from the American Diabetes Association: national cross sectional study. *bmj*. 2020;369
S70. Heidemann C, Du Y, Schubert I, Rathmann W, Scheidt-Nave C. [Prevalence and temporal trend of known diabetes mellitus: results of the German Health Interview and Examination Survey for Adults (DEGS1)]. *Bundesgesundheitsblatt Gesundheitsforschung Gesundheitsschutz*. May 2013;56(5-6):668-77. Pravalenz und zeitliche Entwicklung des bekannten Diabetes mellitus: Ergebnisse der Studie zur Gesundheit Erwachsener in Deutschland (DEGS1). doi:10.1007/s00103-012-1662-5
S71. Tamayo T, Brinks R, Hoyer A, Kuß O, Rathmann W. The prevalence and incidence of diabetes in Germany: an analysis of statutory health insurance data on 65 million individuals from the years 2009 and 2010. *Deutsches Ärzteblatt International*. 2016;113(11):177.
S72. Soriguer F, Goday A, Bosch-Comas A, et al. Prevalence of diabetes mellitus and impaired glucose regulation in Spain: the Di@ bet. es Study. *Diabetologia*. 2012;55:88-93.
S73. NHS Digital. Health Survey for England 2019 December 2020. Accessed September 2022. https://digital.nhs.uk/data-and-information/publications/statistical/health-survey-for-england/2019#data-sets
S74. Prevention CfDCa. *National Diabetes Statistics Report 2022 - Estimates of Diabetes and Its Burden in the United States*. 2022. https://www.cdc.gov/diabetes/pdfs/data/statistics/national-diabetes-statistics-report.pdf
S75. Welfare AIoHa. High blood pressure. Updated 19 Jul 2019. https://www.aihw.gov.au/reports/risk-factors/high-blood-pressure/data
S76. Malta DC, Santos NBd, Perillo RD, Szwarcwald CL. Prevalence of high blood pressure measured in the Brazilian population, National Health Survey, 2013. *Sao Paulo Medical Journal*. 2016;134:163-170.
S77. Zhang M, Shi Y, Zhou B, et al. Prevalence, awareness, treatment, and control of hypertension in China, 2004-18: findings from six rounds of a national survey. *bmj*. 2023;380
S78. Neuhauser H, Thamm M, Ellert U. [Blood pressure in Germany 2008-2011: results of the German Health Interview and Examination Survey for Adults (DEGS1)]. *Bundesgesundheitsblatt Gesundheitsforschung Gesundheitsschutz*. May 2013;56(5-6):795-801. Blutdruck in Deutschland 2008-2011 : Ergebnisse der Studie zur Gesundheit Erwachsener in Deutschland (DEGS1). doi:10.1007/s00103-013-1669-6
S79. Corbatón-Anchuelo A, Martínez-Larrad MT, Prado-González Nd, Fernández-Pérez C, Gabriel R, Serrano-Ríos M. Prevalence, treatment, and associated factors of hypertension in Spain: a comparative study between populations. *International Journal of Hypertension*. 2018;2018
S80. Ostchega Y. *Hypertension Prevalence Among Adults Aged 18 and Over: United States, 2017–2018*. Vol. 364. 2020. https://www.cdc.gov/nchs/data/databriefs/db364-h.pdf
S81. Liew D, Audehm RG, Haikerwal D, et al. Epidemiology of heart failure: Study of Heart failure in the Australian Primary carE setting (SHAPE). *ESC Heart Failure*. 2020;7(6):3871-3880.
S82. Oliveira GMMd, Brant LCC, Polanczyk CA, et al. Cardiovascular statistics–brazil 2020. *Arquivos Brasileiros de Cardiologia*. 2020;115:308-439.
S83. Sun L, Zou L-X, Han Y-C, et al. Forecast of the incidence, prevalence and burden of end-stage renal disease in Nanjing, China to the Year 2025. *BMC nephrology*. 2016;17(1):1-8.
S84. Stork S, Handrock R, Jacob J, et al. Epidemiology of heart failure in Germany: a retrospective database study. *Clin Res Cardiol*. Nov 2017;106(11):913-922. doi:10.1007/s00392-017-1137-7
S85. Dutch Heart Foundation. Cardiovascular disease in the Netherlands, 2021. 2021.
S86. Farré N, Vela E, Clèries M, et al. Real world heart failure epidemiology and outcome: A population-based analysis of 88,195 patients. *PloS one*. 2017;12(2):e0172745.
S87. Tsao CW, Aday AW, Almarzooq ZI, et al. Heart disease and stroke statistics—2022 update: a report from the American Heart Association. *Circulation*. 2022;145(8):e153-e639.
S88. Statistics ABo. Heart, stroke and vascular disease. https://www.abs.gov.au/statistics/health/health-conditions-and-risks/heart-stroke-and-vascular-disease/2017-18#data-downloads
S89. Li X, Wu C, Lu J, et al. Cardiovascular risk factors in China: a nationwide population-based cohort study. *The Lancet Public Health*. 2020;5(12):e672-e681.
S90. Gosswald A, Schienkiewitz A, Nowossadeck E, Busch MA. [Prevalence of myocardial infarction and coronary heart disease in adults aged 40-79 years in Germany: results of the German Health Interview and Examination Survey for Adults (DEGS1)]. *Bundesgesundheitsblatt Gesundheitsforschung Gesundheitsschutz*. May 2013;56(5-6):650-5. Pravalenz von Herzinfarkt und koronarer Herzkrankheit bei Erwachsenen im Alter von 40 bis 79 Jahren in Deutschland: Ergebnisse der Studie zur Gesundheit Erwachsener in Deutschland (DEGS1). doi:10.1007/s00103-013-1666-9

S91. Welfare AIoHa. *Heart, stroke and vascular disease: Australian facts*. 2023. https://www.aihw.gov.au/reports/heart-stroke-vascular-diseases/hsvd-facts/contents/heart-stroke-and-vascular-disease-and-subtypes/stroke
S92. Bensenor IM, Goulart AC, Szwarcwald CL, Vieira MLFP, Malta DC, Lotufo PA. Prevalence of stroke and associated disability in Brazil: National Health Survey-2013. *Arquivos de neuro-psiquiatria*. 2015;73:746-750.
S93. Busch MA, Schienkiewitz A, Nowossadeck E, Gosswald A. [Prevalence of stroke in adults aged 40 to 79 years in Germany: results of the German Health Interview and Examination Survey for Adults (DEGS1)]. *Bundesgesundheitsblatt Gesundheitsforschung Gesundheitsschutz*. May 2013;56(5-6):656-60. Pravalenz des Schlaganfalls bei Erwachsenen im Alter von 40 bis 79 Jahren in Deutschland: Ergebnisse der Studie zur Gesundheit Erwachsener in Deutschland (DEGS1). doi:10.1007/s00103-012-1659-0
S94. Boix R, del Barrio JL, Saz P, et al. Stroke prevalence among the Spanish elderly: an analysis based on screening surveys. *BMC Neurology*. 2006/10/16 2006;6(1):36. doi:10.1186/1471-2377-6-36
S95. NHS Digital. *Health Survey for England - 2011, Health, social care and lifestyles*. 2012. https://digital.nhs.uk/data-and-information/publications/statistical/health-survey-for-england/health-survey-for-england-2011-health-social-care-and-lifestyles
S96. ANZDATA Registry. *45th Report, Chapter 1: Incidence of Kidney Failure with Replacement Therapy. Australia and New Zealand Dialysis and Transplant Registry*. 2022. https://www.anzdata.org.au/wp-content/uploads/2023/02/c01_incidence_2021_ar_2022_v1.0_FINAL.pdf
S97. Luxardo R, Ceretta L, González-Bedat M, Ferreiro A, Rosa-Diez G. The Latin American Dialysis and Renal Transplantation Registry: report 2019. *Clinical Kidney Journal*. 2022;15(3):425-431.
S98. (AstraZeneca) H. *Burden of chronic kidney disease: China (AZ Data on File)*. 2022.
S99. Zhang L, Zhao M-H, Zuo L, et al. China kidney disease network (CK-NET) 2016 annual data report. *Kidney International Supplements*. 2020;10(2):e97-e185.
S100. Frei U, Schober-Halstenberg H. Nierenersatztherapie in Deutschland. *Bericht über dialysebehandlung und nierentransplantation in Deutschland*. 2005:2008.
S101. Escobar EM, de Enfermos Renales RE. The Spanish Renal Registry: 2013 report and evolution from 2007 to 2013. *Nefrología (English Edition)*. 2016;36(2):97-120.
S102. Registro Español de Enfermos Renales (REER). *Registro Español de Enfermos Renales (REER): Informe 2020*. 2020. Accessed April 2023. https://www.senefro.org/contents/webstructure/MEMORIA_REER_2020.pdf
S103. Registry UR. *UK Renal Registry 24th Annual Report– data to 31/12/2020*. 2022. Accessed October 2022. https://ukkidney.org/sites/renal.org/files/publication/file-attachments/24th_UKRR_ANNUAL_REPORT_BOOK%20version%203.pdf
S104. United States Renal Data System. *2022 USRDS Annual Data Report: Epidemiology of kidney disease in the United States*. 2022. https://adr.usrds.org/2022
S105. Australian Bureau of Statistics. Australian Health Survey: Biomedical Results for Chronic Diseases, 2011-12. May 2023, https://www.abs.gov.au/statistics/health/health-conditions-and-risks/australianhealth-survey-biomedical-results-chronic-diseases/latest-release#chronic-kidneydisease.
S106. Piccolli AP, Nascimento MMd, Riella MC. Prevalence of chronic kidney disease in a population in southern Brazil (Pro-Renal Study). *Brazilian Journal of Nephrology*. 2017;39:384-390.
S107. Zhang L, Wang F, Wang L, et al. Prevalence of chronic kidney disease in China: a cross-sectional survey. *The lancet*. 2012;379(9818):815-822.
S108. Girndt M, Trocchi P, Scheidt-Nave C, Markau S, Stang A. The Prevalence of Renal Failure. Results from the German Health Interview and Examination Survey for Adults, 2008-2011 (DEGS1). *Dtsch Arztebl Int*. Feb 12 2016;113(6):85-91. doi:10.3238/arztebl.2016.0085
S109. Digital N. Health Survey for England, 2016. December 2017. Accessed September 2022. https://digital.nhs.uk/data-and-information/publications/statistical/health-survey-for-england/health-survey-for-england-2016#highlights
S110. Otero A, de Francisco A, Gayoso P, García F. Prevalence of chronic renal disease in Spain: Results of the EPIRCE study. Nefrologia [Internet]. 2010 [cited 2013 Sep 3]; 30 (1): 78–86.
S111. Centers for Disease Control and Prevention NCfHS. National Health and Nutrition Examination Survey (NHANES 2017-2020). https://wwwn.cdc.gov/nchs/nhanes/continuousnhanes/default.aspx?Cycle=2017-2020
S112. Nephrology JSo. Clinical practice guidebook for diagnosis and treatment of chronic kidney disease 2012. *Nihon Jinzo Gakkai Shi*. 2012;54(8):1034-1191.
S113. Gorostidi M, Sánchez-Martínez M, Ruilope LM, et al. Chronic kidney disease in Spain: Prevalence and impact of accumulation of cardiovascular risk factors. *Nefrología (English Edition)*. 2018;38(6):606-615.

S114. United States Renal Data System. 2*020 USRDS Annual Data Report: Epidemiology of kidney disease in the United States.* 2020.
S115. Escobar C, Aranda U, Palacios B, et al. Epidemiology, clinical profile, management, and two-year risk complications among patients with chronic kidney disease in Spain. *nefrologia*. 2021;41(6):670-688.
S116. United States Renal Data System. *2022 USRDS Annual Data Report: Epidemiology of kidney disease in the United States*. , 2022. https://adr.usrds.org/2022
S117. United States Renal Data System. *2010 USRDS Annual Data Report: Epidemiology of kidney disease in the United States.* 2010.
S118. Schneider MP, Hilgers KF, Schmid M, et al. Blood pressure control in chronic kidney disease: A cross-sectional analysis from the German Chronic Kidney Disease (GCKD) study. *PloS one*. 2018;13(8):e0202604.
S119. Collins AJ, Foley RN, Herzog C, et al. US renal data system 2010 annual data report. *American Journal of Kidney Diseases*. 2011;57(1):A8.
S120. United States Renal Data System. *2018 USRDS Annual Data Report: Epidemiology of kidney disease in the United States.* 2018.
S121. Yuan J, Zou X-R, Han S-P, et al. Prevalence and risk factors for cardiovascular disease among chronic kidney disease patients: results from the Chinese cohort study of chronic kidney disease (C-STRIDE). *BMC nephrology*. 2017;18(1):1-12.
S122. Beck H, Titze SI, Hübner S, et al. Heart failure in a cohort of patients with chronic kidney disease: the GCKD study. *PloS one*. 2015;10(4):e0122552.
S123. United States Renal Data System. 2018 Annual Data Report: Chronic Kidney Disease (CKD) in the United States. https://www.niddk.nih.gov/about-niddk/strategic-plans-reports/usrds/prior-data-reports/2018
S124. Masson P, Webster AC, Hong M, Turner R, Lindley RI, Craig JC. Chronic kidney disease and the risk of stroke: a systematic review and meta-analysis. *Nephrology Dialysis Transplantation*. 2015;30(7):1162-1169.
S125. Welfare AIoHa. *Incidence of insulin-treated diabetes in Australia*. 2020. https://www.aihw.gov.au/reports/diabetes/incidence-of-insulin-treated-diabetes/data
S126. Centers for Disease Control and Prevention NCfHS. Incidence of Newly Diagnosed Diabetes. https://www.cdc.gov/diabetes/data/statistics-report/newly-diagnosed-diabetes.html
S127. Wang M, Gong W-W, Pan J, et al. Incidence and time trends of type 2 diabetes mellitus among adults in Zhejiang Province, China, 2007-2017. *Journal of Diabetes Research*. 2020;2020
S128. National Diabetes Surveillance at the Robert Koch Institute. *Diabetes in Germany – National Diabetes Surveillance Report 2019*. 2019. Accessed February, 2023. https://diabsurv.rki.de/SharedDocs/downloads/DE/DiabSurv/diabetes-report_2019_eng.pdf?__blob=publicationFile&v=12
S129. Ubink-Veltmaat L, Bilo H, Groenier K, Houweling S, Rischen R, Meyboom-de Jong B. Prevalence, incidence and mortality of type 2 diabetes mellitus revisited: a prospective population-based study in The Netherlands (ZODIAC-1). *European journal of epidemiology*. 2003;18:793-800.
S130. Rojo-Martínez G, Valdés S, Soriguer F, et al. Incidence of diabetes mellitus in Spain as results of the nation-wide cohort di@ bet. es study. *Scientific reports*. 2020;10(1):2765.
S131. Zghebi SS, Steinke DT, Carr MJ, Rutter MK, Emsley RA, Ashcroft DM. Examining trends in type 2 diabetes incidence, prevalence and mortality in the UK between 2004 and 2014. *Diabetes, Obesity and Metabolism*. 2017;19(11):1537-1545.
S132. Dannenberg AL, Garrison RJ, Kannel WB. Incidence of hypertension in the Framingham Study. *American Journal of Public Health*. 1988;78(6):676-679.
S133. Liang Y, Liu R, Du S, Qiu C. Trends in incidence of hypertension in Chinese adults, 1991–2009: the China Health and Nutrition Survey. *International journal of cardiology*. 2014;175(1):96-101.
S134. Beunza JJ, Martínez-González MÁ, Serrano-Martínez M, Alonso Á. Incidence of hypertension in a cohort of Spanish university graduates: the SUN study. *Revista Española de Cardiología (English Edition)*. 2006;59(12):1331-1334.
S135. Wang H, Chai K, Du M, et al. Prevalence and incidence of heart failure among urban patients in China: a national population-based analysis. *Circulation: Heart Failure*. 2021;14(10):e008406.
S136. Fernández-Gassó L, Hernando-Arizaleta L, Palomar-Rodriguez JA, Abellán-Pérez MV, Hernandez-Vicente A, Pascual-Figal DA. Population-based study of first hospitalizations for heart failure and the interaction between readmissions and survival. *Revista Española de Cardiología (English Edition)*. 2019;72(9):740-748.
S137. Khera R, Pandey A, Ayers CR, et al. Contemporary epidemiology of heart failure in fee-for-service Medicare beneficiaries across healthcare settings. *Circulation: Heart Failure*. 2017;10(11):e004402.

S138. Nedkoff LJ, Briffa TG, Preen DB, et al. Age-and sex-specific trends in the incidence of hospitalized acute coronary syndromes in Western Australia. *Circulation: Cardiovascular Quality and Outcomes*. 2011;4(5):557-564.
S139. Mefford MT, Li BH, Qian L, et al. Sex-specific trends in acute myocardial infarction within an integrated healthcare network, 2000 through 2014. *Circulation*. 2020;141(7):509-519.
S140. Kaptoge S, Pennells L, De Bacquer D, et al. World Health Organization cardiovascular disease risk charts: revised models to estimate risk in 21 global regions. *The Lancet Global Health*. 2019;7(10):e1332-e1345.
S141. Kramer C, Meisinger C, Kirchberger I, et al. Epidemiological trends in mortality, event rates and case fatality of acute myocardial infarction from 2004 to 2015: results from the KORA MI registry. *Ann Med*. Dec 2021;53(1):2142-2152. doi:10.1080/07853890.2021.2002926
S142. Forcadell MJ, Vila-Córcoles A, de Diego C, Ochoa-Gondar O, Satué E. Incidence and mortality of myocardial infarction among Catalonian older adults with and without underlying risk conditions: The CAPAMIS study. *European Journal of Preventive Cardiology*. 2018;25(17):1822-1830.
S143. de Santana NM, dos Santos Figueiredo FW, de Melo Lucena DM, et al. The burden of stroke in Brazil in 2016: an analysis of the Global Burden of Disease study findings. *BMC research notes*. 2018;11(1):1-5.
S144. Stahmeyer JT, Stubenrauch S, Geyer S, Weissenborn K, Eberhard S. The Frequency and Timing of Recurrent Stroke: An Analysis of Routine Health Insurance Data. *Dtsch Arztebl Int*. Oct 18 2019;116(42):711-717. doi:10.3238/arztebl.2019.0711
S145. Vega T, Zurriaga O, Ramos JM, et al. Stroke in Spain: epidemiologic incidence and patterns; a health sentinel network study. *Journal of Stroke and Cerebrovascular diseases*. 2009;18(1):11-16.
S146. Madsen TE, Khoury JC, Leppert M, et al. Temporal trends in stroke incidence over time by sex and age in the GCNKSS. *Stroke*. 2020;51(4):1070-1076.
S147. Hall MJ, Levant S, DeFrances CJ. *Hospitalization for congestive heart failure: United States, 2000-2010*. US Department of Health and Human Services, Centers for Disease Control and …; 2012.
S148. Tseng CH. Clinical features of heart failure hospitalization in younger and elderly patients in Taiwan. *European journal of clinical investigation*. 2011;41(6):597-604.
S149. Lawson CA, Zaccardi F, Squire I, et al. 20-year trends in cause-specific heart failure outcomes by sex, socioeconomic status, and place of diagnosis: a population-based study. *The Lancet Public Health*. 2019;4(8):e406-e420.
S150. Meems L, van Veldhuisen D, de Boer R. Progress in heart failure management in the Netherlands and beyond: long-term commitment to deliver high-quality research and patient care. *Netherlands Heart Journal*. 2020;28:31-38.
S151. Anguita Sánchez M, Bonilla Palomas JL, García Márquez M, Bernal Sobrino JL, Elola Somoza FJ, Marín Ortuño F. Temporal trends in hospitalization and in-hospital mortality rates due to heart failure by age and sex in Spain (2003-2018). 10.1016/j.rec.2021.04.017. *Revista Española de Cardiología (English Edition)*. 2021;74(11):993-996. doi:10.1016/j.rec.2021.04.017
S152. Lin C-Y, Hsieh M-C, Kor C-T, Hsieh Y-P. Association and risk factors of chronic kidney disease and incident diabetes: a nationwide population-based cohort study. *Diabetologia*. 2019;62:438-447.
S153. Lin E, Chertow GM, Yan B, Malcolm E, Goldhaber-Fiebert JD. Cost-effectiveness of multidisciplinary care in mild to moderate chronic kidney disease in the United States: A modeling study. *PLoS medicine*. 2018;15(3):e1002532.
S154. Zelnick LR, Weiss NS, Kestenbaum BR, et al. Diabetes and CKD in the United States population, 2009–2014. *Clinical Journal of the American Society of Nephrology*. 2017;12(12):1984-1990.
S155. Hounkpatin HO, Harris S, Fraser SD, et al. Prevalence of chronic kidney disease in adults in England: comparison of nationally representative cross-sectional surveys from 2003 to 2016. *BMJ open*. 2020;10(8):e038423.
S156. Crews DC, Plantinga LC, Miller III ER, et al. Prevalence of chronic kidney disease in persons with undiagnosed or prehypertension in the United States. *Hypertension*. 2010;55(5):1102-1109.
S157. United States Renal Data System. *Annual Data Report: Atlas of Chronic Kidney Disease and End-Stage Renal Disease in the United States*. 2009. Accessed August 2023. https://www.niddk.nih.gov/about-niddk/strategic-plans-reports/usrds/prior-data-reports
S158. Bansal N, Zelnick L, Bhat Z, et al. Burden and outcomes of heart failure hospitalizations in adults with chronic kidney disease. *Journal of the American College of Cardiology*. 2019;73(21):2691-2700.
S159. Health AIo, Welfare. Acute kidney injury in Australia: a first national snapshot. AIHW Canberra; 2015.
S160. Sawhney S, Bell S, Black C, et al. Harmonization of epidemiology of acute kidney injury and acute kidney disease produces comparable findings across four geographic populations. *Kidney International*. 2022;101(6):1271-1281.

S161. Liu B-C, Tang R-N, Liu Z-H. Current clinical research of acute kidney injury in China. *Chinese Medical Journal*. 2015;128(09):1268-1271.
S162. Matrisch L, Karsten H, Schucke J, Rau Y. Increase in Registered Acute Kidney Injuries in German Hospitals. *Cureus*. Mar 2023;15(3):e36868. doi:10.7759/cureus.36868
S163. Grams ME, Sang Y, Ballew SH, et al. A meta-analysis of the association of estimated GFR, albuminuria, age, race, and sex with acute kidney injury. *American Journal of Kidney Diseases*. 2015;66(4):591-601.
S164. Duarte TTP, Magro MCS. Recovery of Renal Function in Clinical Patients with Acute Kidney Injury: Impact on Mortality. *Life*. 2022;12(6):852.
S165. Zealand TTSoAaN. *Clinical Guidelines for Organ Transplantation from Deceased Donors*. 2022. https://tsanz.com.au/storage/documents/TSANZ_Clinical_Guidelines_Version-110_Final.pdf
S166. Hecking M, Tu C, Zee J, et al. Sex-specific differences in mortality and incident dialysis in the chronic kidney disease outcomes and practice patterns study. *Kidney International Reports*. 2022;7(3):410-423.
S167. Han Y-C, Huang H-M, Sun L, et al. Epidemiological study of RRT-treated ESRD in Nanjing-a ten-year experience in nearly three million insurance covered population. *PloS one*. 2016;11(2):e0149038.
S168. ANZDATA Registry. *ANZDATA 43rd Annual Report 2020 (Data to 2019)*. 2020. https://www.anzdata.org.au/report/anzdata-43rd-annual-report-2020-data-to-2019/.
S169. Lugon JR, Gordan PA, Thomé FS, et al. A web-based platform to collect data from ESRD patients undergoing dialysis: methods and preliminary results from the Brazilian dialysis registry. *International Journal of Nephrology*. 2018;2018
S170. Statistia. Total number of patients active on the organ transplant waiting list in Germany from 2019 to 2021, by organ. https://www.statista.com/statistics/538312/number-of-patients-active-on-organ-transplant-waiting-list-in-germany/
S171. Crespo M, Mazuecos A, Domínguez-Gil B. Global perspective on kidney transplantation: Spain. *Kidney360*. 2021;2(11):1840.
S172. Statista. Total number of patients on the organ transplant waiting list in Spain from 2017 to 2021, by organ. Updated 2019. https://www.statista.com/statistics/538386/number-of-patients-active-on-organ-transplant-waiting-list-in-spain/
S173. Chinese National Renal Data System. 国家肾脏病医疗质量控制中心. Accessed 2022. http://www.cnrds.net
S174. Nefrovisie. *RENINE annual report 2022*. 2022. https://www.nefrovisie.nl/wp-content/uploads/2023/02/Jaarrapport_rapportagejaar2021.pdf
S175. Liu Y, Wang L, Han X, et al. The profile of timing dialysis initiation in patients with end-stage renal disease in China: a cohort study. *Kidney and Blood Pressure Research*. 2020;45(2):180-193.
S176. Chan CT, Blankestijn PJ, Dember LM, et al. Dialysis initiation, modality choice, access, and prescription: conclusions from a Kidney Disease: Improving Global Outcomes (KDIGO) Controversies Conference. *Kidney Int*. Jul 2019;96(1):37-47. doi:10.1016/j.kint.2019.01.017
S177. Chan CT, Blankestijn PJ, Dember LM, et al. Dialysis initiation, modality choice, access, and prescription: conclusions from a Kidney Disease: Improving Global Outcomes (KDIGO) Controversies Conference. *Kidney international*. 2019;96(1):37-47.
S178. Camacho X, Nedkoff L, Wright FL, et al. Relative contribution of trends in myocardial infarction event rates and case fatality to declines in mortality: an international comparative study of 1· 95 million events in 80· 4 million people in four countries. *The Lancet Public Health*. 2022;7(3):e229-e239.
S179. Brant LC, Passaglia LG. High Mortality for Myocardial Infarction in Latin America and the Caribbean: Making the Case for Systems of Care Implementation in Brazil. *Arquivos Brasileiros de Cardiologia*. 2022;119:979-980.
S180. Ferreira GMTdM, Correia LC, Reis H, et al. Increased mortality and morbidity due to acute myocardial infarction in a public hospital, in Feira de Santana, Bahia. *Arquivos Brasileiros de Cardiologia*. 2009;93:97-104.
S181. Long Z, Liu W, Zhao Z, et al. Case Fatality Rate of Patients with Acute Myocardial Infarction in 253 Chest Pain Centers—China, 2019–2020. *China CDC Weekly*. 2022;4(24):518.
S182. Asaria P, Bennett JE, Elliott P, et al. Contributions of event rates, pre-hospital deaths, and deaths following hospitalisation to variations in myocardial infarction mortality in 326 districts in England: a spatial analysis of linked hospitalisation and mortality data. *The Lancet Public Health*. 2022;7(10):e813-e824.
S183. Jones NR, Roalfe AK, Adoki I, Hobbs FR, Taylor CJ. Survival of patients with chronic heart failure in the community: a systematic review and meta‐analysis. *European journal of heart failure*. 2019;21(11):1306-1325.

S184. Pérez G, Pena A, Sala J, et al. Acute myocardial infarction case fatality, incidence and mortality rates in a population registry in Gerona, Spain, 1990–1992. *International journal of epidemiology*. 1998;27(4):599-604.
S185. Thrift AG, Dewey HM, Macdonell RA, McNeil JJ, Donnan GA. Stroke incidence on the east coast of Australia: the north east Melbourne stroke incidence study (NEMESIS). *Stroke*. 2000;31(9):2087-2092.
S186. Minelli C, Fu Fen L, Camara Minelli DP. Stroke incidence, prognosis, 30-day, and 1-year case fatality rates in Matão, Brazil: a population-based prospective study. *Stroke*. 2007;38(11):2906-2911.
S187. He F, Blackberry I, Yao L, Xie H, Rasekaba T, Mnatzaganian G. Pooled incidence and case-fatality of acute stroke in Mainland China, Hong Kong, and Macao: A systematic review and meta-analysis. *Plos one*. 2022;17(6):e0270554.
S188. Koton S, Schneider AL, Rosamond WD, et al. Stroke incidence and mortality trends in US communities, 1987 to 2011. *Jama*. 2014;312(3):259-268.
S189. Vaartjes I, O'Flaherty M, Capewell S, Kappelle J, Bots M. Remarkable decline in ischemic stroke mortality is not matched by changes in incidence. *Stroke*. 2013;44(3):591-597.
S190. Díaz-Guzmán J, Egido J-A, Gabriel-Sánchez R, Barberá-Comes G, Fuentes-Gimeno B, Fernández-Pérez C. Stroke and transient ischemic attack incidence rate in Spain: the IBERICTUS study. *Cerebrovascular diseases*. 2012;34(4):272-281.
S191. Centers for Disease Control and Prevention NCfHS. Underlying Cause of Death, 2018-2021, Single Race Results. Accessed February 16, 2023, https://wonder.cdc.gov/controller/datarequest/D158;jsessionid=C2DBD5ACD4B2658CAD174386E4BF#Citation
S192. Bendall A, Tan S, See E, Fazio T, Toussaint N. INCIDENCE AND EPIDEMIOLOGY OF ACUTE KIDNEY INJURY (AKI) AT AN AUSTRALIAN METROPOLITAN QUATERNARY REFERRAL CENTRE. WILEY 111 RIVER ST, HOBOKEN 07030-5774, NJ USA; 2021:33-33.
S193. Dos Santos RP, Carvalho ARdS, Peres LAB. Incidence and risk factors of acute kidney injury in critically ill patients from a single centre in Brazil: a retrospective cohort analysis. *Scientific Reports*. 2019;9(1):1-8.
S194. Inda-Filho AJ, Ribeiro HS, Vieira EA, Ferreira AP. Epidemiological profile of acute kidney injury in critically ill patients admitted to intensive care units: A Prospective Brazilian Cohort. *Brazilian Journal of Nephrology*. 2021;43:580-585.
S195. Kidneys T. *Reporting the rate of Acute Kidney Injury (AKI) within England – the current state of the NHS AKI Master Patient Index and Registry*. 2018. https://www.thinkkidneys.nhs.uk/aki/wp-content/uploads/sites/2/2018/03/Reporting-the-rate-of-AKI-January-2018.pdf
S196. Yang L, Xing G, Wang L, et al. Acute kidney injury in China: a cross-sectional survey. *The Lancet*. 2015;386(10002):1465-1471.
S197. Xu X, Nie S, Liu Z, et al. Epidemiology and clinical correlates of AKI in Chinese hospitalized adults. *Clinical Journal of the American Society of Nephrology*. 2015;10(9):1510-1518.
S198. Khadzhynov D, Schmidt D, Hardt J, et al. The Incidence of Acute Kidney Injury and Associated Hospital Mortality: A Retrospective Cohort Study of Over 100 000 Patients at Berlin‘s Charité Hospital. *Deutsches Ärzteblatt International*. 2019;116(22):397.
S199. Uhel F, Peters-Sengers H, Falahi F, et al. Mortality and host response aberrations associated with transient and persistent acute kidney injury in critically ill patients with sepsis: a prospective cohort study. *Intensive care medicine*. 2020;46:1576-1589.
S200. Sohaney R, Yin H, Shahinian V, et al. In-hospital and 1-year mortality trends in a national cohort of US veterans with acute kidney injury. *Clinical Journal of the American Society of Nephrology*. 2022;17(2):184-193.
S201. Teng T-HK, Finn J, Hobbs M, Hung J. Heart failure: incidence, case fatality, and hospitalization rates in Western Australia between 1990 and 2005. *Circulation: Heart Failure*. 2010;3(2):236-243.
S202. Fernandes AD, Fernandes GC, Mazza MR, et al. A 10-year trend analysis of heart failure in the less developed Brazil. *Arquivos brasileiros de cardiologia*. 2020;114:222-231.
S203. Feng Y, Chen X, Schaufelberger M, Zhang Q, Fu M. Patient-level comparison of heart failure patients in clinical phenotype and prognosis from China and Sweden. *BMC Cardiovascular Disorders*. 2022;22(1):1-8.
S204. Taylor CJ, Ordóñez‐Mena JM, Jones NR, et al. National trends in heart failure mortality in men and women, United Kingdom, 2000–2017. *European journal of heart failure*. 2021;23(1):3-12.
S205. Wyld ML, Wyburn KR, Chadban SJ. Global perspective on kidney transplantation: Australia. *Kidney360*. 2021;2(10):1641.
S206. Foresto RD, Pestana JOM, Silva Jr HT. Brasil: the leading public kidney transplant program worldwide. SciELO Brasil; 2020. p. 708-709.
S207. Zhang Z, Liu Z, Shi B. Global perspective on kidney transplantation: China. *Kidney360*. 2022;3(2):364.

S208. Mehrabi A, Wiesel M, Zeier M, et al. Results of renal transplantation using kidneys harvested from living donors at the University of Heidelberg. *Nephrology Dialysis Transplantation*. 2004;19(suppl_4):iv48-iv54.
S209. Ojo AO, Morales JM, González-Molina M, et al. Comparison of the long-term outcomes of kidney transplantation: USA versus Spain. *Nephrology Dialysis Transplantation*. 2013;28(1):213-220.
S210. Levey AS, De Jong PE, Coresh J, et al. The definition, classification, and prognosis of chronic kidney disease: a KDIGO Controversies Conference report. *Kidney international*. 2011;80(1):17-28.
S211. Statistics ABo. Deaths, Australia. Updated 2021. https://www.abs.gov.au/statistics/people/population/deaths-australia/2021
S212. Platform WD. Deaths by sex and age group for a selected country or area and year. https://platform.who.int/mortality/themes/theme-details/MDB/all-causes
S213. Zhu J, Cui L, Wang K, et al. Mortality pattern trends and disparities among Chinese from 2004 to 2016. *BMC Public Health*. 2019;19:1-7.
S214. Statistisches Bundesamt (Destatis). Population: Germany, reference date, age, nationality/gender/marital status. Updated 2022. Accessed April 2023, https://www-genesis.destatis.de/genesis/online?operation=table&code=12411-0006&bypass=true&levelindex=0&levelid=1678288674953#abreadcrumb
S215. Estadistica INd. Mortality (Spanish/foreign) by age (year by year) and sex. Accessed April 2023, https://www.ine.es/jaxiT3/Datos.htm?t=27153
S216. Duffin E. Death rate by age and sex in the U.S. 2019. Accessed February 16, 2023, https://www.statista.com/statistics/241572/death-rate-by-age-and-sex-in-the-us/
S217. Wyld M, Lee C, Zhuo X, et al. Cost to government and society of chronic kidney disease stage 1–5: a national cohort study. *Internal medicine journal*. 2015;45(7):741-747.
S218. Wu J, Yang L. The economic burden of chronic kidney disease in China. *Value in Health*. 2013;16(3):A181.
S219. Gandjour A, Armsen W, Wehmeyer W, Multmeier J, Tschulena U. Costs of patients with chronic kidney disease in Germany. *PLoS One*. 2020;15(4):e0231375. doi:10.1371/journal.pone.0231375
S220. van Oosten MJ, Logtenberg SJ, Leegte MJ, et al. Age-related difference in health care use and costs of patients with chronic kidney disease and matched controls: analysis of Dutch health care claims data. *Nephrology Dialysis Transplantation*. 2020;35(12):2138-2146.
S221. Pons R, Torregrosa E, Hernández-Jaras J, et al. El coste del tratamiento farmacológico en la enfermedad renal crónica. *Nefrología*. 2006;26(3):358-364.
S222. Lorenzo-Sellares V, Pedrosa MI, Santana-Expósito B, García-González Z, Barroso-Montesinos M. Análisis de costes y perfil sociocultural del enfermo renal: Impacto de la modalidad de tratamiento. *Nefrología (Madrid)*. 2014;34(4):458-468.
S223. Golestaneh L, Alvarez PJ, Reaven NL, et al. All-cause costs increase exponentially with increased chronic kidney disease stage. *The American journal of managed care*. 2017;23(10 Suppl):S163-S172.
S224. Cass A, S.J. Chadban, M. Gallagher,. The economic impact of end-stage kidney disease in Australia: Projections to 2020. https://www.georgeinstitute.org/publications/the-economic-impact-of-end-stagekidney-disease-in-australia-projections-to-2020
S225. Improvement N. *Healthcare costing standards for England Acute: Costing methods*. 2019. https://www.england.nhs.uk/wp-content/uploads/2020/08/Acute_CMs_standards20.pdf
S226. Howard K, White S, Salkeld G, et al. Cost‐effectiveness of screening and optimal management for diabetes, hypertension, and chronic kidney disease: a modeled analysis. *Value in health*. 2010;13(2):196-208.
S227. Silva SB, Caulliraux HM, Araújo CAS, Rocha E. Uma comparação dos custos do transplante renal em relação às diálises no Brasil. *Cadernos de Saúde Pública*. 2016;32:e00013515.
S228. Wang F, Yang C, Long J, et al. Executive summary for the 2015 Annual Data Report of the China Kidney Disease Network (CK-NET). *Kidney Int*. Mar 2019;95(3):501-505. doi:10.1016/j.kint.2018.11.011
S229. Zhao W, Zhang L, Han S, et al. Cost analysis of living donor kidney transplantation in China: a single-center experience. *Ann Transplant*. Apr-Jun 2012;17(2):5-10. doi:10.12659/aot.883217
S230. Shukri A, Mettang T, Scheckel B, et al. Hemodialysis and Peritoneal Dialysis in Germany from a Health Economic View-A Propensity Score Matched Analysis. *Int J Environ Res Public Health*. Oct 27 2022;19(21):14007. doi:10.3390/ijerph192114007
S231. Kleophas W, Reichel H. International study of health care organization and financing: development of renal replacement therapy in Germany. *Int J Health Care Finance Econ*. Sep 2007;7(2-3):185-200. doi:10.1007/s10754-007-9020-0
S232. Mohnen SM, van Oosten MJ, Los J, et al. Healthcare costs of patients on different renal replacement modalities–analysis of Dutch health insurance claims data. *PLoS One*. 2019;14(8):e0220800.

S233. Sanidad Md. Hospital discharge records in the national health system. Accessed February 2022, https://www.sanidad.gob.es/en/estadEstudios/estadisticas/cmbdhome.htm.
S234. Arrieta J, Rodríguez-Carmona A, Remón C, et al. La diálisis peritoneal es la mejor alternativa coste-efectiva para la sostenibilidad del tratamiento con diálisis. *Nefrología (Madrid)*. 2011;31(5):505-513.
S235. England N, Improvement N. National Cost Collection 2019. NHS England and NHS Improvement. https://www. england. nhs. uk/wp-content …; 2020.
S236. England N. *Service Specifications*. 2017. https://www.england.nhs.uk/wp-content/uploads/2017/05/service-spec-adult-kidney-transplant-service.pdf
S237. System USRD. *2020 USRDS Annual Data Report: Epidemiology of kidney disease in the United States*. , 2020. https://adr.usrds.org/2020
S238. Elbasha E, Greaves W, Roth D, Nwankwo C. Cost‐effectiveness of elbasvir/grazoprevir use in treatment‐naive and treatment‐experienced patients with hepatitis C virus genotype 1 infection and chronic kidney disease in the United States. *Journal of viral hepatitis*. 2017;24(4):268-279.
S2439. Axelrod DA, Schnitzler MA, Xiao H, et al. An economic assessment of contemporary kidney transplant practice. *American Journal of Transplantation*. 2018;18(5):1168-1176.
S240. Cooper JT, Lloyd A, Sanchez JJG, Sörstadius E, Briggs A, McFarlane P. Health related quality of life utility weights for economic evaluation through different stages of chronic kidney disease: a systematic literature review. *Health and Quality of Life Outcomes*. 2020;18:1-11.
S241. van Haalen, H., Jackson, J., Spinowitz, B., Milligan, G., & Moon, R. Impact of chronic kidney disease and anemia on health-related quality of life and work productivity: analysis of multinational real-world data. *BMC Nephrology*. 2020;21(1). doi: 10.1186/s12882-020-01746-4
S242. Eriksson D, Karlsson L, Eklund O, et al. Real-world costs of autosomal dominant polycystic kidney disease in the Nordics. *BMC health services research*. 2017;17(1):1-9.
S243. AstraZeneca Data on File. Environmental burden by stage and treatment modality. 2023.
